# Administration of vitamin D and its metabolites in critically ill adult patients: an updated systematic review with meta-analysis of randomized controlled trials

**DOI:** 10.1186/s13054-022-04139-1

**Published:** 2022-09-06

**Authors:** Johannes Menger, Zheng-Yii Lee, Quirin Notz, Julia Wallqvist, M. Shahnaz Hasan, Gunnar Elke, Martin Dworschak, Patrick Meybohm, Daren K. Heyland, Christian Stoppe

**Affiliations:** 1 Department of Anesthesiology, Intensive Care, Emergency and Pain Medicine, University Hospital Würzburg, Würzburg, Germany; 2 Department of Anesthesiology, Faculty of Medicine, University of Malaya, Kuala Lumpur, Malaysia; 3 Department of Anesthesiology, University Hospital Rheinisch-Westfälische Technische Hochschule Aachen, Aachen, Germany; 4 Department of Anesthesiology and Intensive Care Medicine, University Medical Center Schleswig-Holstein, Campus Kiel, Kiel, Germany; 5 Division of Cardiothoracic and Vascular Anesthesia and Intensive Care Medicine, Department of Anesthesia, Intensive Care Medicine and Pain Medicine, Medical University of Vienna, Vienna, Austria; 6 Clinical Evaluation Research Unit, Department of Critical Care Medicine, Queen’s University, KGH Research Institute, Kingston Health Sciences Centre, Kingston, ON Canada

**Keywords:** Vitamin D, Critically ill, Nutrition, Meta-analysis, Mortality, Mechanical ventilator weaning

## Abstract

**Background:**

The clinical significance of vitamin D administration in critically ill patients remains inconclusive. The purpose of this systematic review with meta-analysis was to investigate the effect of vitamin D and its metabolites on major clinical outcomes in critically ill patients, including a subgroup analysis based on vitamin D status and route of vitamin D administration.

**Methods:**

Major databases were searched through February 9, 2022. Randomized controlled trials of adult critically ill patients with an intervention group receiving vitamin D or its metabolites were included. Random-effect meta-analyses were performed to estimate the pooled risk ratio (dichotomized outcomes) or mean difference (continuous outcomes). Risk of bias assessment included the Cochrane tool for assessing risk of bias in randomized trials.

**Results:**

Sixteen randomized clinical trials with 2449 patients were included. Vitamin D administration was associated with lower overall mortality (16 studies: risk ratio 0.78, 95% confidence interval 0.62–0.97, *p* = 0.03; *I*^2^ = 30%), reduced intensive care unit length of stay (12 studies: mean difference − 3.13 days, 95% CI − 5.36 to − 0.89, *n* = 1250, *p* = 0.006; *I*^2^ = 70%), and shorter duration of mechanical ventilation (9 studies: mean difference − 5.07 days, 95% CI − 7.42 to − 2.73, *n* = 572, *p* < 0.0001; *I*^2^ = 54%). Parenteral administration was associated with a greater effect on overall mortality than enteral administration (test of subgroup differences, *p* = 0.04), whereas studies of parenteral subgroups had lower quality. There were no subgroup differences based on baseline vitamin D levels.

**Conclusions:**

Vitamin D supplementation in critically ill patients may reduce mortality. Parenteral administration might be associated with a greater impact on mortality. Heterogeneity and assessed certainty among the studies limits the generalizability of the results.

*Trial registration*: PROSPERO international prospective database of systematic reviews (CRD42021256939—05 July 2021).

**Supplementary Information:**

The online version contains supplementary material available at 10.1186/s13054-022-04139-1.

## Background

Vitamin D and its metabolites are secosteroid hormones [1] known for their key role in regulating calcium-phosphorus homeostasis and bone metabolism. More recently, the cardiovascular- and more importantly immuno-modulating functions of vitamin D became of increasing interest [1, 2].

Inflammation, oxidative stress and immune dysfunction are important factors in the pathogenesis of critical illness, which may ultimately lead to organ dysfunction [3]. In these patients, significantly reduced vitamin D (25-hydroxyvitamin D) serum levels are frequent and independently associated with higher incidence and severity of sepsis [4, 5]. A recent meta-analysis highlighted the association between vitamin D deficiency in critically ill patients with sepsis and unfavorable outcomes [6].

Amrein et al. [7] found first signals that high-dose enteral vitamin D supplementation could be of clinical relevance especially in those critically ill patients with a vitamin D deficiency. The subsequent multi-center placebo-controlled (VIOLET) study investigating high dose enteral vitamin D supplementation in vitamin D deficient critically ill patients however did not reveal better clinical outcomes as compared to placebo [8]. On the other hand, a recent small randomized controlled trial (RCT) demonstrated lower mortality among patients after parenteral substitution of vitamin D [9].

Given the high interest in this topic with demonstrated survival benefits after vitamin D administration and the following high research activity this systematic review and meta-analysis aimed to provide an updated evaluation of the impact of vitamin D supplementation on clinical outcomes in critically ill patients. Particular emphasis was put on the effect of vitamin D supplementation in relevant subgroups including vitamin D deficiency and route of administration.

## Methods

This systematic review with meta-analysis of RCTs was conducted following the 2020 Preferred Reporting Items for Systematic reviews and Meta-Analyses (PRISMA) statement (see Additional file 1 for PRISMA checklist) [10].

### Eligibility criteria

RCTs had to meet all of the following criteria to be included: (1) critically ill adult patients (age ≥ 18 years). Critically ill is defined as being treated in intensive care unit (ICU) environment, i.e., either mechanically ventilated or if this cannot be determined, a mortality of > 5%; (2) either enteral or parenteral administration of vitamin D or a vitamin D metabolite; (3) compared with “standard care” or a predefined “control group”; (4) clinically important outcomes including one of the following: mortality, length of—ICU and hospital—stay (LOS), or duration of mechanical ventilation must have been reported in the RCT. Primary outcome in this meta-analysis was overall mortality. If > 1 type of mortality was reported, we selected in the order of 28-day, hospital, ICU and other mortality. Secondary outcomes were hospital and ICU LOS and duration of mechanical ventilation.

Studies of patients undergoing elective surgery or studies that addressed only biochemical, metabolic, or nutritional outcomes were excluded. Unpublished manuscripts and conference abstracts were not eligible for inclusion. No language restrictions were defined. No studies were excluded based on the date of publication.

### Information sources

We systematically searched the following databases from database launch to February 9, 2022: MEDLINE, EMBASE and CENTRAL (Cochrane Database of Systematic Reviews and the Cochrane Central Register of Controlled Trials) through OVID, and CINAHL (Cumulative Index to Nursing and Allied Health Literature) through EBSCOhost. In addition, we searched for additional articles from published systematic reviews [11–14], personal records, contacts and ClinicalTrials.gov for ongoing studies.

### Search strategy and selection process

The search was conducted with 3 major concepts: (1) critically ill patients, (2) vitamin D and its metabolites and (3) established search filter for RCT [15], adult and human (OVID expert search). Each concept was searched by using subject headings and relevant keywords that were combined with the Boolean operator “OR.” The concepts were then combined with Boolean operator “AND.” Examples of the subject headings or keywords used are "critical care," "critical illness," "vitamin D," "25 hydroxyvitamin D," "ergocalciferol" and "cholecalciferol." The detailed search strategy and selection process is presented in Additional file 1.

### Data collection process

A standardized form was used for data collection, which was completed independently by two reviewers (JM and MSH). Data collection of the Chinese article was done by ZYL. A third reviewer resolved discrepancies (ZYL). Corresponding authors were asked for additional information in cases published articles did not report complete outcome data. No assumption or data conversion was made if we were unable to obtain this information.

### Data items

Data was extracted regarding study characteristics (including risk of bias assessment), patients’ characteristics (including baseline vitamin D status), type of intervention (including route of administration of vitamin D) and outcome (mortality [overall, 28-day, hospital, ICU], ICU and hospital LOS, duration of mechanical ventilation).

### Study quality and risk of bias assessment

Two independent authors critically appraised an included study using an established methodological quality scoring system for risk of bias assessment. This scoring system ranges from 0 to 14 points (higher score indicates higher study quality). This quality assessment tool has been used in prior critical care nutrition systematic reviews and allows for comparisons of quality across topics and across time [16–18]. A third author resolved any disagreement. A trial is considered a level 1 study if all 3 of the following criteria were fulfilled: (1) concealed randomization, (2) double-blinded (outcome adjudication must be blinded) and (3) conducted an intention-to-treat analysis. If any of the above characteristics are not met, the study will be classified as a level 2 study. Additional file 1 provides further information on risk of bias assessment.

### Synthesis methods and effect measures

The risk ratio (RR) with 95% confidence interval (CI) was used for binary outcomes and mean difference with 95% CI was used for continuous outcomes.

We grouped the studies by route of administration of vitamin D for subgroup analysis in enteral/per os (EN/PO) and intravenous/intramuscular (IV/IM). In 3 studies, data from the two interventional groups were pooled for the meta-analyses [19–21]. In the EN/PO versus IV/IM subgroup analysis, the IM and EN subgroups of Hansanloei [20] were analyzed by splitting the control group based on the recommendation by the Cochrane Handbook [22].

Meta-analysis was conducted using RevMan 5.4 (Cochrane IMS, Oxford, UK). For dichotomized outcomes, the pooled RR was estimated by the DerSimonian and Laird random effect meta-analysis. For continuous outcomes, the random effect mean difference was estimated. Heterogeneity was quantified by the I^2^ measure. The result of the meta-analysis is presented in the forest plot generated by RevMan. Publication bias was evaluated by funnel plot. Egger’s test for funnel plot asymmetry was performed by using the metafor package in RStudio (version 1.3.1093) if ≥ 10 studies were included in the meta-analysis. Subgroup analysis were conducted for studies that used EN/PO or IV/IM to substitute vitamin D and for studies randomizing only vitamin D deficient (< 30 ng/mL at baseline) patients. We defined all methodology (including statistics, subgroups and risk of bias analysis) before the start of data extraction for this study unless stated otherwise.

A post-hoc subgroup analysis of the studies was performed with regard to the subgroup single versus multicenter studies. A multicenter study was defined as a study conducted in > 1 hospital. If the study was conducted in > 1 ICUs but in the same hospital, it was considered as a single-center study. Post-hoc two reviewers (JM and MSH) assessed version 2 of the Cochrane tool for assessing risk of bias in randomized trials (RoB 2) [23]. ZYL assessed the Chinese article. A third reviewer resolved discrepancies (ZYL). Post-hoc Trial Sequential Analysis with a priori defined assumptions was performed for the primary outcome result (overall mortality) to analyze the risk of type 1 and type 2 error due to repetitive testing of accumulating data in this meta-analysis [24, 25]. We made the following assumptions to construct the trial sequential monitoring boundaries: risk of type 1 error of 5%, risk of type 2 error of 20%, relative risk reduction of 20% and heterogeneity of 50%. An α-spending adjusted CI was calculated. To assess the certainty in the evidence and the strength of recommendation we used post-hoc the GRADE approach [26, 27].

A *p* value < 0.05 was considered significant and values  > 0.05 but < 0.20 were considered a trend towards significance (for hypothesis-generating purpose).

## Results

### Study selection

**Fig. 1:**
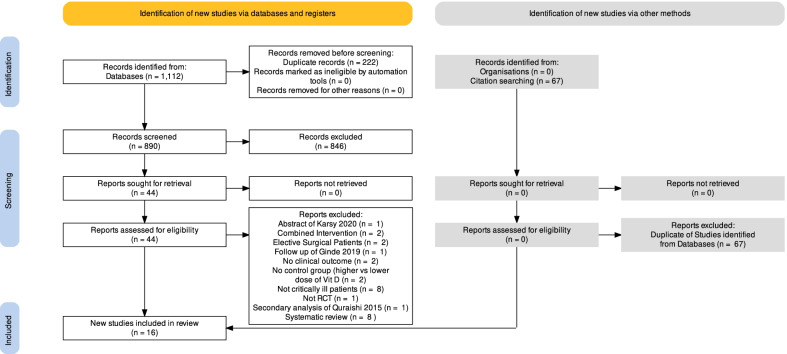
PRISMA 2020 flow diagram depicting the study selection process

We identified a total of 1112 records from MEDLINE (*n* = 135), EMBASE (*n* = 686), CENTRAL (*n* = 91), CINAHL (*n* = 200). Using Covidence, 222 duplicates were removed. After title and abstract screening, 44 studies were retrieved in full text and assessed for eligibility. Two authors independently reviewed the eligibility of the studies (JM and ZYL), and these were confirmed by two senior authors (DKH and CS). Twenty-eight studies were deemed ineligible (see details in Additional file 1). Finally, 16 studies were included. The citations of previous systematic review and meta-analysis were also searched. No additional studies were identified as all potentially relevant citations were contained in the database search. The study selection process is shown in the PRISMA 2020 flow diagram (Fig. 1). We identified two active studies from ClinicalTrials.gov (see Additional file 1). We obtained additional data upon request from the corresponding authors: ICU LOS [28, 29], hospital LOS [28], duration of mechanical ventilation [28, 29].

### Study characteristics

A total of 2449 patients were enrolled in 16 RCTs (sample size range 24–1059) [7–9, 19–21, 28–37] Twelve studies included only vitamin D deficient patients (< 30 ng/mL) [7–9, 20, 30, 32–37] Four studies did not have a threshold vitamin D level for inclusion [19, 21, 28, 31]. Across all studies, vitamin D was administered through PO, EN, IV or IM route. In 8 studies, vitamin D was administered EN/PO [7, 8, 19, 21, 28, 30, 36, 37], whereas in 7 studies vitamin D was administered IV/IM [9, 29, 31–35]. All but one study compared vitamin D versus placebo, whereas Hasanloei compared EN vitamin D versus IM vitamin D versus standard care [20]. Study characteristics are shown in Table 1.

**Table 1:** Analyzed randomized controlled trials: study population, type of intervention, methodological scoring

Author, year	N	Population	Vitamin D status	EN/NG	IV/IM	Methodological quality score	Quality level
Amrein et al. 2011[30]	25	Mixed ICU patients, expected LOS > 48 h	25-hydroxyvitamin D ≤ 20 ng/ml	Single dose of 540 000 IU NG		11	1
Amrein et al. 2014[7]	492	Medical and surgical ICU patients, expected LOS > 48 h	25-hydroxyvitamin D ≤ 20 ng/ml	Dose of 540 000 IU NG. 28 days after first dose, 5 monthly maintenance doses of 90 000 IU		12	1
Leaf et al. 2014[31]	67	ICU patients with severe sepsis/septic shock	Mixed		2 µg calcitriol, single dose IV 2ug	12	1
Quraishi et al. 2015[21]	30	ICU patients with sepsis	Mixed	Single dose of 200 000 IU or 400 000 IU		12	1
Han et al. 2016 [19]	31	Mixed ICU patients receiving EN, expected LOS 96 h	Mixed	50 000 IU daily for 5 days or 100 000 IU daily for 5 days		8	1
Ding et al. 2017 [32]	57	Septic patients with ICU stay > 48 h	25-hydroxyvitamin D < 30 ug/mL		Single dose of 300,000 IU IM	7	2
Miroliaee et al. 2018[33]	51	Mechanically ventilated ICU patients with pneumonia	25-hydroxyvitamin D < 30 ng/ml		Single dose of 300,000 IU IM	6	2
Ginde et al. 2019[8]	1360	ICU patients with > 1 risk factors for death or lung injury	25-hydroxy vitamin D < 20 ng/ml	Single dose of 540 000 IU via NG		9	2
Hasanloei et al. 2019[20]	72	Mechanically ventilated (> 48 h) trauma ICU patients with LOS ≥ 7d	25-hydroxy vitamin D 10–30 ng/ml	(1) 50 000 IU daily for five days via NG	(2) Single dose of 300,000 IU IM	5	2
Miri et al. 2019 [35]	44	Mechanically ventilated ICU patients	"Vitamin D deficient"		Single dose of 300,000 IU IM	5	2
Yousefian et al. 2019 [34]	66	Mechanically ventilated stroke patients	25-hydroxy vitamin D < 20 ng/ml		300,000 IU IM up to 3 times per week	7	2
Ingels et al. 2020 [29]	24	ICU patients, expected LOS > 10d	25-hydroxy vitamin D < 10 ng/ml		200 µg calcidiol loading dose on admission followed by 15 µg daily for 10 days	7	2
Karsy et al. 2020 [36]	274	Neurocritical care patients	25-hydroxy vitamin D < 20 ng/ml	Single dose of 540 000 IU		13	1
Sharma et al. 2020 [37]	35	Mechanically ventilated patients with traumatic brain injury	"Vitamin D deficient"	Single dose of 120 000 IU via feeding tube		8	1
Sistanizad et al. 2021 [9]	30	Mechanically ventilated ICU patients surviving the first 72 h	25-hydroxy vitamin D < 10 ng/ml		300,000 IU IM up to 3 times per wk	4	2
Bhattacharyya et al. 2021 [28]	126	Sepsis patients expected to survive > 96 h with EN access	Mixed	Single dose of 540,000 IU dissolved in 45 ml of milk		11	2

*ICU* intensive care unit, *LOS* length of stay, *EN* enteral, *NG* nasogastric, *IV* intravenous, *IM* intramuscular, *IU* international units of vitamin D3 (cholecalciferol; if not stated otherwise), Methodological quality score ranging from 0 to 14 (higher score indicates higher study quality), Quality level: In level 1 all of the following criteria were fulfilled: (1) concealed randomization, (2) double-blinded (outcome adjudication must be blinded) and (3) conducted an intention-to-treat analysis. If any of the above characteristics are not met, the study will be classified as a level 2 study

### Risk of bias in studies

Seven RCTs [7, 19, 21, 30, 31, 36, 37] were rated as level 1 and 9 RCTs [8, 9, 20, 28, 29, 32–35] as level 2 study. The median methodological quality scoring was 8 (IQR [range] 6.75–11.25 [4-13]; Table 1). RoB 2 plots can be found in Additional file 1.

### Mortality

**Fig. 2:**
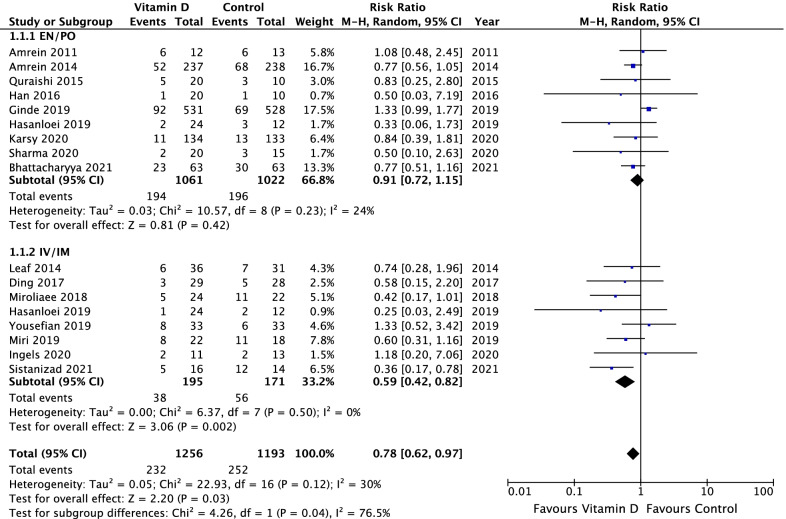
Overall mortality in critically ill patients: vitamin D compared to placebo (or standard of care) including subgroup analysis of route of administration

All studies reported about mortality. In comparison to placebo, vitamin D supplementation significantly reduced the overall mortality (RR 0.78, 95% CI 0.62–0.97, *p* = 0.03, *I*^2^ = 35%, Fig. 2). Nine studies reported 28-day mortality [7–9, 21, 31–33, 35, 36]. There was a trend observed with vitamin D supplementation on 28-day mortality compared to placebo (RR 0.73, 95% CI 0.52–1.01, *p* = 0.06, *I*^2^ = 57%, Additional file 2). Six studies [7, 8, 19, 29–31] reported hospital mortality and 3 studies [7, 29, 31] reported ICU mortality. Vitamin D supplementation had no significant effect on these two outcomes (Additional file 3 and Additional file 4).

Trial sequential analysis for overall mortality results is provided in Additional file 1. For overall mortality results, the cumulative Z-curve neither crosses the predefined trial sequential monitoring boundaries nor enters the futility area. Trial sequential analysis yielded a required information size of 5426 patients. The pooled effect with α-spending adjusted CI was 0.77 (0.54–1.11).

### Length of ICU and hospital stay

**Fig. 3:**
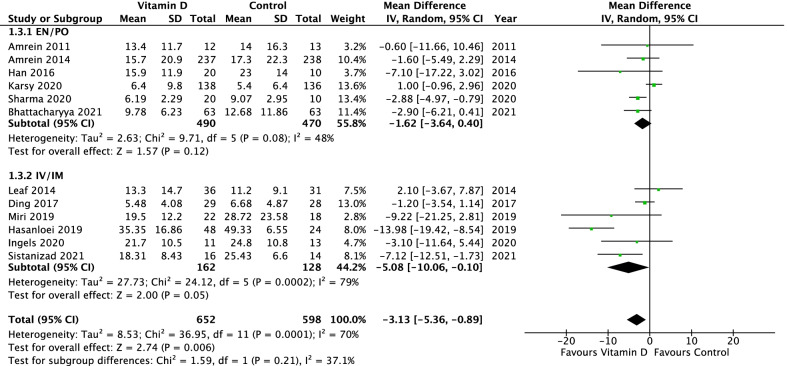
ICU length of stay in critically ill patients: vitamin D compared to placebo (or standard of care) including subgroup analysis of route of administration

Twelve studies [7, 9, 19, 20, 28–32, 35–37] reported ICU LOS. In comparison to placebo, vitamin D supplementation was associated with a reduction in ICU LOS (mean difference − 3.13 days, 95% CI − 5.36 to − 0.89, *p* = 0.006, *I*^2^ = 70%, Fig. 3). Seven studies [7, 8, 19, 28, 30, 31, 36] reported hospital LOS with vitamin D supplementation having no significant effect (Additional file 5).

### Duration of mechanical ventilation

**Fig. 4:**
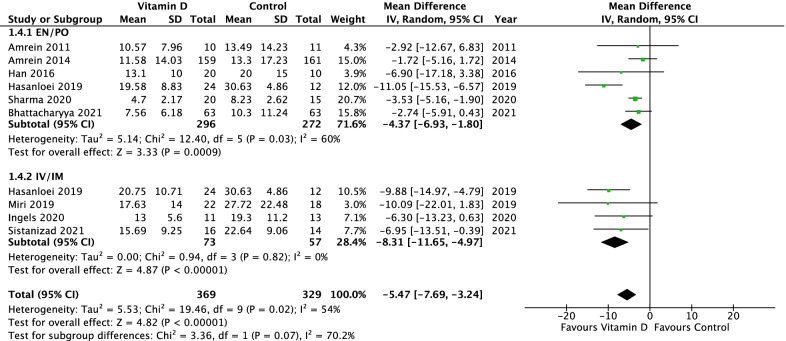
Duration of mechanical ventilation in critically ill patients: vitamin D compared to placebo (or standard of care) including subgroup analysis of route of administration

Nine studies [7, 9, 19, 20, 28–30, 35, 37] reported duration of mechanical ventilation. In comparison to placebo, vitamin D supplementation was associated with a significant reduction in mechanical ventilation duration (mean difference − 5.07 days, 95% CI − 7.42 to − 2.73; *p* < 0.0001, *I*^2^ = 54%, Fig. 4).

### Subgroup IV/IM versus EN/PO

The studies using IV/IM as route of administration showed a significantly reduced overall mortality in comparison to EN/PO route of administration (RR 0.59, 95% CI 0.42–0.82, vs. RR 0.90, 95% CI 0.71–1.15; *p* = 0.04, *I*^2^ = 76%; Fig. 2). In the subgroup of IV/IM vitamin D administration, a significantly lower 28-day mortality compared to placebo and a significant subgroup difference compared to EN/PO intake was observed (RR 0.51, 95% CI 0.35–0.74, *p* = 0.0005, *I*^2^ = 0% vs. RR 0.97, 95% CI 0.67–1.40, *p* = 0.86, *I*^2^ = 56%; *p* = 0.02, *I*^2^ = 82.3%, Additional file 2). There was no significant subgroup difference regarding ICU or hospital mortality (Additional file 6 and Additional file 7). The subgroup analysis revealed neither a significantly shorter duration of mechanical ventilation (Chi^2^ = 3.36, *p* = 0.07, *I*^2^ = 70%; Fig. 4) nor a significant shorter ICU or hospital LOS (Fig. 3; Additional file 8) for IV/IM administration.

### Subgroup vitamin D < 30 ng/mL versus no threshold

There was neither any significant subgroup difference in any studied mortality (Additional file 3, 4, 9 and 10), nor in ICU and hospital LOS (Additional file 5 and Additional file 11) between studies without a threshold vitamin D level at baseline compared to vitamin D levels < 30 ng/mL at baseline. There was no significant subgroup difference regarding duration of mechanical ventilation (Additional file 12).

### Reporting bias

To assess reporting bias, funnel plots are provided for all analyzed outcomes in Additional file 1. Eggers' tests for outcomes with 10 or more studies did not indicate the presence of funnel plot asymmetry for overall mortality (-0.735, 95% CI − 1.61 to 0.14, *p* = 0.12) or ICU length of stay (-1.562, 95% CI − 3.41 to 0.29, *p* = 0.13).

### GRADE evaluation

Following the GRADE approach, the certainty of our findings regarding the primary outcome overall mortality are rated as low. Serious risk of bias and serious imprecision lead to a downgrading. Certainty of our findings regarding the secondary outcomes ICU/hospital LOS and duration of mechanical ventilation are rated as low. A “GRADE evidence profile” is provided in Additional file 1.

### Single versus multicenter studies

Post-hoc subgroup analysis of the studies with regard to the subgroup single- versus multicenter studies showed that all but four studies [8, 19, 31, 33] were single-center studies. No significant subgroup difference was found between single and multicenter with regard to mortality, ICU LOS and duration of mechanical ventilation (Additional file 1).

## Discussion

To our knowledge, this is the first systematic review and meta-analysis of completed RCTs reporting evidence that vitamin D administration in critically ill patients may significantly affect multiple major clinical outcomes with lower overall mortality, shorter ICU LOS and duration of mechanical ventilation, particularly when vitamin D was administered via the IV/IM route.

### Relation to previous studies and meta-analysis

The findings of this study are in contrast to two major studies comprising a total of 1.553 patients that have been published recently and are part of our meta-analysis [7, 8]. The VITdAL-ICU trial [7] for example failed to find significant differences in hospital LOS or hospital mortality in the overall patient population. It, however, demonstrated a benefit in those patients with severe vitamin D deficiency. The VIOLET trial included over 1000 adult critically ill patients with low vitamin D (25(OH)D < 20 ng/ml) not showing a difference between the placebo group and the vitamin D group, while a one-time ultra-high enteral loading dose (540,000 IU) was given without a maintenance dose [8]. In fact, this approach demonstrated to be inefficient in a large meta-analysis for the prevention of respiratory infections, while daily or weekly vitamin D showed a strong protective effect, especially in patients with severe vitamin D deficiency [38, 39]. Although a recent analysis by Lan et al. including 9 RCTs showed no significant difference in the pooled analysis for 28-day mortality, ICU, hospital LOS, and mechanical ventilation, the authors concede that the heterogeneous populations may have diluted potential signals of benefits after vitamin D administration [11].

### Potential effects of vitamin D in specific cohorts of critically ill patients

Our meta-analysis supports the notion that time of mechanical ventilation and ICU LOS is reduced after vitamin D supplementation in the subgroup with vitamin D deficient patients (< 30 ng/mL). In addition, overall and 28-day mortality showed a trend towards reduced mortality in the vitamin D deficient group after supplementation compared to placebo. Upon ICU admission, the majority of patients have significantly reduced 25-hydroxyvitamin D levels, which remain significantly reduced over the entire ICU LOS [7, 8]. The VITdAL-ICU and VIOLET RCTs both showed severe vitamin D deficiency (< 20 ng/mL) in the majority of recruited patients. The trial by Amrein et al. indicated [7] that the subgroup with further depleted vitamin D levels (< 12 ng/mL) might benefit most from high-dose vitamin D supplementation.

### Potential explanations for received findings

We found seven studies investigating the effect of IM/IV vitamin D supplementation. When comparing IV/IM vitamin D application with placebo, we detected an association with a significantly shorter time of mechanical ventilation, reduced ICU LOS as well as a reduced 28 day and overall mortality. Gupta et al. further found significantly higher 25(OH)D levels in healthy subjects receiving the IM application form compared to PO supplementation of cholecalciferol [40]. Hasanloei did not find a significant difference regarding 25(OH)D levels between these groups; however, they described a limited bioavailability of vitamin D PO compared to the IM application. Lan et al. [11] did not find any differences in clinical outcomes related to the route of administration, whereas they only included three studies investigating the effect of IV/IM vitamin D supplementation as these were the only ones available at that time.

It is important to note that current evidence only provides limited data about the effectiveness of vitamin D supplementation and the route of administration. In the majority of the included RCTs, cholecalciferol, the inactive form of vitamin D, was administered EN/PO. Since ICU patients frequently suffer from organ dysfunction, it remains unclear if this inactive form of vitamin D is adequately absorbed by the gastro-intestinal tract of the critically ill patient and adequately converted into its biological active form by liver and kidneys. In hematopoietic stem cell transplant patients, for example, vitamin D levels between 30 and 60 ng/L could only be reached in 64% of patients despite an “aggressive” enteral dosage regimen [41]. Therefore, it needs to be elucidated if the supplemented vitamin D dose as well as the product type is appropriate to correct low baseline levels. Insufficient supplementation might be one reason for the lack of efficacy. Only 3 studies [7, 30, 31] reported about calcitriol levels, the active metabolite of vitamin D and resulting effects. In the majority of the trials, it is unknown if and when active vitamin D levels were high enough to unfold their beneficial biological effects at all. Recently, serum 1,25(OH)2D levels have been investigated in cardiac surgery patients [42]. Higher levels were associated with less organ dysfunction, shorter hospital stay and lower inflammatory marker levels, indicating the high clinical relevance of this biological active form. Furthermore, the multinational multicenter VITDALIZE trial [43] currently evaluates a new promising supplementation strategy. Enrolled critically ill patients with severe vitamin D deficiency (25-hydroxyvitamin D ≤ 12 ng/mL) will receive in addition to the loading dose of 540,000 IU cholecalciferol after ICU admission a daily dose of 4000 IU for 90 days. The resulting clinical outcomes will be compared to such patients that received placebo.

Bioavailability of the investigated product might be a crucial pillar in the challenging task of supplementing vitamin D in critically ill patients correctly. These considerations are supported by our findings that the effect of vitamin D supplementation on clinical outcome seems to depend on the administration strategy and route of administration of vitamin D. Current research investigates the effects of administration of the pharmaceutical form of 25(OH)D, calcifediol and active vitamin D or analogues in critically ill patients [29, 44]. With these forms, the consequences of impaired absorption and activation of native vitamin D may be reduced in critically ill patients. Patients with intestinal malfunction, fat malabsorption and poor liver function may benefit more from supplementation with 25(OH)D. The absorption of this form does not depend on fat absorption or the formation of chylomicrons, does not require hepatic hydroxylation and might lead to a faster and proportionally higher increment in plasma 25(OH)D per unit given compared with vitamin D.

### The role of dosing and timing

Despite the promising signals for parenteral and intramuscular vitamin D, it needs to be acknowledged that data about its safety at different doses remains still unclear and thus urgently needs to be addressed in future studies. A parenteral high dose native vitamin D may be of special relevance for patients with severe vitamin D deficiency not responsive to oral vitamin D supplementation, whereas comparable doses are currently only available as intramuscular injection [38]. Yet, it has to be critically acknowledged that the intramuscular injection may be associated with more complications and even contraindicated in patients being on anticoagulation. Therefore, oral calcifediol [25(OH)D] may represent a promising alternative as it has a high rate of intestinal absorption with important advantages in case of decreased intestinal absorption capacity [45].

Apart from the appropriate form of vitamin D supplementation, the timing of administration might be of major relevance, too. Metabolization of inactive vitamin D3 into the active form takes 2–3 days. The presumed impact of active vitamin D in this patient cohort is thought to be largely based on reduction in inflammatory marker in the acute critical phase of illness; late administration might not translate into clinically meaningful effects. The included studies showed different time windows in which the interventional strategy was started after ICU admission. While the VIOLET trial [8] and the study by Bhattacharyya et al. [28] required supplementation within the first 14 h after ICU admission, the VITdal-ICU [7] as well as the RECTIFY study allowed cholecalciferol intake within the first two days after ICU admission. In the smaller studies (< 100 patients in total), the timeframe varied up to 72 h after ICU admission [20] or was not reported at all.

During the SARS-CoV-2 pandemic, vitamin D gained remarkable attention, as several observational trials found an association between vitamin status and the risk to develop COVID-19 and especially a severe clinical course of the disease [46]. The potential protective effects of vitamin D on COVID-19 related health outcomes are stipulated to be mediated (1) modulating the cytokine storm (2) modulating neutrophil activity, (3) maintaining the pulmonary epithelial barrier, and (4) stimulating epithelial repair [47]. In order to investigate the effect of vitamin D supplementation in these vulnerable SARS-CoV-2 positive patients, a systematic Cochrane review has been published in 2021 [48]. Until then, 3 RCTs have been included at the time, whereas over 21 trials were still ongoing [49]. Consequently, pooling of data was not possible. When comparing the included studies, results showed that administration of the more active form, calcifediol, reduced the need for ICU treatment of patients requiring hospitalization due to proven COVID-19 compared to placebo [50]. It is important to note that calcifediol has not been tested in the general critically ill population yet.

### Study limitations

The low certainty of evidence for the primary outcome overall mortality assessed with the GRADE approach suggests that further research using high quality RCTs is needed to strengthen these findings. The incorporation of studies with low to moderate methodological quality and inconsistent results in our meta-analysis remains a weakness. For overall mortality results of the trial sequential analysis cannot confirm the positive result of this meta-analysis. Consequently, this imprecision led to a downgrading of the certainty of our findings in the GRADE approach. Therefore, the observed effects in favor of vitamin D administration have to be considered cautiously and require more research as heterogeneity adjusted required information size has not been reached yet and the line of futility has not been crossed. It is well known that meta-analyses are often triggered by an effect in one of the preceding primary studies; a finding that may be false-significant [51]. Inclusion of primary studies with false-significant effects may lead to biased effect estimates and inflated type I error rates, which needs to be carefully considered in the interpretation of the received results.

Some limitations have to be considered cautiously for the interpretation of the received findings. First, the demonstrated heterogeneity among the studies may limit the generalizability of the received data. Second the included studies showed high variance with respect to the sample size, with several smaller studies that contributed to the overall results. Third, as outlined before, the difference observed in the administration strategy, risk of bias and different patient populations makes it challenging to make strong conclusions and therefore the received certainty of evidence is low. More specific research in this area is urgently needed and encouraged.

Particularly, outcomes for IV/IM administration of vitamin D are derived from seven studies with all but one ranking below the median methodological quality score. Therefore, the chance to introduce bias is high in this analysis as no trial in this subgroup included more than 67 patients. Although no significant subgroup different was seen between single and multicenter studies, most evidence seems to come from single-center studies with a higher risk of bias. Studies reporting duration of mechanical ventilation or ICU LOS do not appear to have systematical accounted for the effect of censoring due to death when calculating mean durations of mechanical ventilation or ICU LOS. Therefore, there might be some residual bias related to these variables.

Nevertheless, the strength of our meta-analysis lies in the usage of several methods to reduce bias (i.e., comprehensive literature search, duplicate data abstraction, and inclusion of non-English-language articles), as well as the personal contact to authors of enclosed trials to obtain additional non-published data.

## Conclusion

The results of this systematic review and meta-analysis suggest that vitamin D supplementation may be associated with reduced overall mortality in critically ill patients. Parenteral administration appears to be related to an accentuated effect. Moreover, vitamin D supplementation might be linked to a significantly reduced duration of ventilation and ICU LOS. As several smaller and inconsistent studies with an inherent risk of bias are part of this analysis, larger and more definitive trials are required to support our findings regarding the type of supplementation and specific patient populations that benefit the most from this intervention.

## Supplementary Information

**Additional file 1.** I Material and Methods: Risk of bias assessment form, II Material and Methods: Search strategy, III Material and methods: PRISMA checklist, IV Results: RoB 2 results, V Results: TSA for overall mortality, VI Results: Funnel plots with legends, VII Results: Excluded studies, VIII Results: Registered ongoing studies, IX Results: Subgroups single vs multi-center; X Results: GRADE evidence profile.

**Additional file 2.** Mortality 28-day in critically ill patients: Vitamin D compared to placebo (or standard of care) including subgroup analysis of route of administration.

**Additional file 3.** Hospital mortality: Vitamin D compared to placebo or standard of care for critically ill patients including subgroup analysis of baseline vitamin D.

**Additional file 4.** ICU mortality: Vitamin D compared to placebo or standard of care for critically ill patients including subgroup analysis of baseline vitamin D.

**Additional file 5.** ICU length of stay: Vitamin D compared to placebo or standard of care for critically ill patients including subgroup analysis of baseline vitamin D.

**Additional file 6.** Hospital mortality: Vitamin D compared to placebo or standard of care for critically ill patients including subgroup analysis of route of administration.

**Additional file 7.** ICU mortality: Vitamin D compared to placebo or standard of care for critically ill patients including subgroup analysis of route of administration.

**Additional file 8.** Hospital length of stay: Vitamin D compared to placebo or standard of care for critically ill patients including subgroup analysis of route of administration.

**Additional file 9.** Overall mortality: Vitamin D compared to placebo or standard of care for critically ill patients including subgroup analysis of baseline vitamin D.

**Additional file 10.** 28-day mortality: Vitamin D compared to placebo or standard of care for critically ill patients including subgroup analysis of baseline vitamin D.

**Additional file 11.** Hospital length of stay: Vitamin D compared to placebo or standard of care for critically ill patients including subgroup analysis of baseline vitamin D.

**Additional file 12**. Duration of mechanical ventilation: Vitamin D compared to placebo or standard of care for critically ill patients including subgroup analysis of baseline vitamin D.

## Data Availability

Data generated or analyzed for this study are included in this published article and its supplementary files. Further information is available from the authors on reasonable request.

## Abbreviations

RCT: Randomized controlled trial
ICU: Intensive care unit
LOS: Length of stay
RR: Risk ratio
CI: Confidence interval
EN: Enteral
PO: Per os/oral (administration)
IV: Intravenous (administration)
IM: Intramuscular (administration)
IQR: Interquartile range
