# Supplementary material for: Administration of vitamin D and its metabolites in critically ill adult patients: an updated systematic review with meta-analysis of randomized controlled trials
Source: Crit Care. 2022 Sep 6;26:268. doi: 10.1186/s13054-022-04139-1 (PMC9446655; doi:10.1186/s13054-022-04139-1)
Supplement: Supplementary file 1 — Additional file 1. I Material and Methods: Risk of bias assessment form, II Material and Methods: Search strategy, III Material and methods: PRISMA checklist, IV Results: RoB 2 results, V Results: TSA for overall mortality, VI Results: Funnel plots with legends, VII Results: Excluded studies, VIII Results: Registered ongoing studies, IX Results: Subgroups single vs multi-center; X Results: GRADE evidence profile. [file 13054_2022_4139_MOESM1_ESM.docx]

Supplemental Material to “Vitamin D supplementation in critically ill adult patients: An updated systematic review with meta-analysis of randomized controlled trials” by Menger J et al.

I Material and Methods: Risk of bias assessment form

II Material and Methods: Search strategy

III Material and methods: PRISMA checklists

IV Results: RoB 2 results

V Results: TSA for overall mortality

VI Results: Funnel plots with legends

VII Results: Excluded studies

VIII Results: Registered ongoing studies

IX Results: Subgroups single vs multi-center

X Results: GRADE evidence profile

I Material and Methods: Risk of bias assessment form

We used the following form to assess the risk of bias/methodological quality:

**Methodological Quality Scoring System**

|  |  | | | | | | |
| --- | --- | --- | --- | --- | --- | --- | --- |
|  | **Score** | | | | | | |
|  | **0** | | **1** | | | **2** | |
| Randomization |  | | Not concealed or not sure |  | | Concealed* randomization |  |
| Analysis | Other |  |  | | | Intention to treat |  |
| Blinding | Not blinded |  | Single blinded  *Check who was blinded:*  Health Care Professionals  Outcomes Assessors | |  | Double blinded |  |
| Patient selection | Selected patients or unable to tell |  | Consecutive eligible patients | |  |  | |
| Comparability of groups at baseline | No or not sure |  | Yes | |  |  | |
| Extent of follow-up | < 100% |  | 100% | |  |  | |
| Treatment protocol | Poorly described |  | Reproducibly described | |  |  | |
| Co-interventions** | Not described |  | Described but not equal or not sure | |  | Well described and all equal |  |
| Outcomes | Not described |  | Partially described | |  | Objectively defined |  |

**Total Score:** **(max 14)**

* Concealed randomization means the person enrolling the patients is unaware of the next treatment

assignment (e.g., phone in randomization, computer generated).

** Extent to which antibiotics, TPN, ventilation, oxygen, transfusions, etc. were applied equally across groups

II Material and Methods: Search strategy

**CONCEPT 1: Filter for RCT, Human and Adults**

| **MEDLINE** | **EMBASE** | **CINAHL** |
| --- | --- | --- |
| 1 Randomized controlled trials as Topic/ | Clinical trial/ | S1 MH randomized controlled trials |
| 2 Randomized controlled trial/ | Randomized controlled trial/ | S2 MH double‐blind studies |
| 3 Random allocation/ | Randomization/ | S3 MH single‐blind studies |
| 4 Double blind method/ | Single blind procedure/ | S4 MH random assignment |
| 5 Single blind method/ | Double blind procedure/ | S5 MH pretest‐posttest design |
| 6 Clinical trial/ | Crossover procedure/ | S6 MH cluster sample |
| 7 Exp Clinical Trials as Topic/ | Placebo/ | S7 TI (randomised OR randomized) |
| 8 Or/1-7 | Randomi?ed controlled trial$.tw. | S8 AB (random*) |
| 9 (clinic$ adj trial$1).tw. | Rct.tw. | S9 TI (trial) |
| 10 ((singl$ or doubl$ or treb$ or tripl$) adj (blind$3 or mask$3)).tw. | Random allocation.tw. | S10 MH (sample size) AND AB (assigned OR allocated OR control) |
| 11 Placebos/ | Randomly allocated.tw. | S11 MH (placebos) |
| 12 Placebo$.tw. | Allocated randomly.tw. | S12 PT (randomized controlled trial) |
| 13 Randomly allocated.tw. | (allocated adj2 random).tw. | S13 AB (control W5 group) |
| 14 (allocated adj2 random).tw. | Single blind$.tw. | S14 MH (crossover design) OR MH (comparative studies) |
| 15 Or/9-14 | Double blind$.tw. | S15 AB (cluster W3 RCT) |
| 16 8 or 15 | ((treble or triple) adj (blind$)).tw. | S16 MH animals+ |
| 17 Case report.tw. | Placebo$.tw. | S17 MH (animal studies) |
| 18 Letter/ | Prospective study/ | S18 TI (animal model*) |
| 19 Historical article/ | Or/1-18 | S19 S16 OR S17 OR S18 |
| 20 Review of reported cases.pt. | Case study/ | S20 MH (human) |
| 21 Review, multicase.pt. | Case report.tw. | S21 S19 NOT S20 |
| 22 Or/17-21 | Abstract report/ or letter/ | S22 S1 OR S2 OR S3 OR S4 OR S5 OR S6 OR S7 OR S8 OR S9 OR S10 OR S11 OR S12 OR S13 OR S14 OR S15 |
| 23 16 not 22 | Or/20-22 | S23 S22 Not S21 |
|  | 19 not 23 |  |
| Children | |  |
| 24 (exp adolescent/ or exp child/ or exp infant/ or (infant disease* or childhood disease*).ti,ab,kf. or (adolescen* or babies or baby or boy? or boyfriend or boyhood or girlfriend or girlhood or child* or girl? or infan* or juvenil* or kid? or minors or minors* or neonat* or neo-nat* or newborn* or new-born* or paediatric* or peadiatric* or pediatric* or perinat* or preschool* or puber* or pubescen* or school* or teen* or toddler? or underage? or under-age? or youth*).ti,ab,kf. or (pediatric* or paediatric* or infan* or child* or adolescen* or young).jn,jw. or (pediatric* or paediatric* or infan* or child* or adolescen* or young).in.) not exp adult/ | (exp adolescence/ or exp adolescent/ or exp child/ or exp childhood disease/ or exp infant disease/ or (adolescen* or babies or baby or boy? or boyfriend or boyhood or girlfriend or girlhood or child* or girl? or infan* or juvenil* or juvenile* or kid? or minors or minors* or neonat* or neo-nat* or neo-nat* or newborn* or new-born* or paediatric* or peadiatric* or pediatric* or perinat* or preschool* or puber* or pubescen* or school or school child* or school* or schoolchild* or schoolchild*).ti,ab,kw. or (pediatric* or paediatric* or infan* or child* or adolescen* or young).jn,jw. or (pediatric* or paediatric* or infan* or child* or adolescen* or young).in. or (teen* or toddler? or underage? or under-age? or youth*).ti,ab,kw.) not exp adult/ | S24 ( (MH "Child+") or (MH "Adolescence") ) NOT (MH "Adult+")  S25 S23 not S24 |
| 25 23 not 24 | 26 24 not 25 |  |
| Animal | |  |
| 26 (Animals/ or Models, Animal/ or Disease Models, Animal/) not Humans/ | 27 (animal or animals or canine* or dog or dogs or feline or hamster* or lamb or lambs or mice or monkey or monkeys or mouse or murine or pig or pigs or piglet* or porcine or primate* or rabbit* or rats or rat or rodent* or sheep* or veterinar*).ti,kw,dq,jx. not (human* or patient*).mp |  |
| 27 ((animal or animals or canine* or dog or dogs or feline or hamster* or lamb or lambs or mice or monkey or monkeys or mouse or murine or pig or pigs or piglet* or porcine or primate* or rabbit* or rats or rat or rodent* or sheep* or veterinar*) not (human* or patient*)).ti,kf,jw. | 28 (exp animal/ or exp juvenile animal/ or adult animal/ or animal cell/ or animal tissue/ or nonhuman/ or animal experiment/ or animal model/) not human/ |  |
| 28 26 or 27 | 29 27 or 28 |  |
| 29 25 not 28 | 30 26 not 29 |  |

NOTE:

- OVID expert search for RCT (https://tools.ovid.com/ovidtools/expertsearches.html) (retrieved Dec 2021)

- OVID expert search for children(broad)(https://tools.ovid.com/ovidtools/expertsearches.html) (retrieved Dec 2021)

- McGill University animal exlusion (https://www.muhclibraries.ca/training-and-guides/excluding-animal-studies) (retrieved Dec 2021)

- CINAHL: Box 3.f Cochrane CINAHL Plus filter

**CONCEPT 2: Critically Ill Population**

| **MeSH Terms** | **Emtree** | **CINAHL** | **Keywords** |
| --- | --- | --- | --- |
| Critical care/  (Note: synonymous with intensive care)  Critical illness/ | Intensive care/  critical illness/  critically ill patient/ | (MH "Critical Care")  (MH "Critical Illness")  (MH "Critically Ill Patients") | critical care.mp.  intensive care.mp.  critical illness.mp.  critically ill.mp. |
| Intensive care units/  burn units/ | intensive care unit/  burn unit/  medical intensive care unit/  neurological intensive care unit/  surgical intensive care unit/ | (MH "Intensive Care Units") | burn unit*.mp. |
| Exp shock/  (includes: multiple organ failure, cardiogenic/ hemorrhagic/ surgical/ trauma shock, Systemic Inflammatory Response Syndrome, cytokine release syndrome, septic shock)  sepsis | Exp shock/  (includes capillary leak syndrome, cardiogenic shock, dengue shock syndrome, experimental shock, hemorrhagic shock, hypovolemic shock, septic shock, toxic shock syndrome, traumatic shock)  systemic inflammatory response syndrome/  sepsis/  septic shock/  septicemia/  urosepsis/  multiple organ failure/ | (MH "Shock+")  (includes: Shock, Cardiogenic Shock, Hemorrhagic Shock, Septic Shock, Surgical Shock, Traumatic, systemic inflammatory response syndrome, cytokine release syndrome)  (MH "Sepsis") | Shock.mp.  Systemic inflammatory response syndrome.ti,kw.  sepsis.mp. septic shock.mp.  multiple organ dysfunction syndrome.mp.  multiple organ failure.mp.  cytokine release syndrome.ti,kw. |
| Respiratory Distress Syndrome/ | respiratory distress syndrome/  acute lung injury/  adult respiratory distress syndrome/  transfusion related acute lung injury/ | (MH "Respiratory Distress Syndrome")  (MH "Respiratory Distress Syndrome, Acute")  (MH "Acute Lung Injury+") | respiratory distress syndrome.mp.  acute lung injury.mp. |
| Burns/ | burn/  burn shock/ | (MH "Burns")  (MH "Burn Units")  (MH "Burn Patients") | (burn$ adj3 patient$).ti,kw. |
| Multiple Trauma/ | Multiple Trauma/ | (MH "Multiple Trauma") | Multi* Trauma*.ti,kw. Multitrauma.ti,kw.  Polytrauma.ti,kw. |
| Pancreatitis, Acute Necrotizing/ | pancreatitis/  acute pancreatitis/ | (MH "Pancreatitis, Acute Necrotizing") | Acute Necroti?ing Pancreatitis.ti,kw. |
| brain injuries/  brain injuries, traumatic/ | brain injury/  traumatic brain injury/ | (MH "Brain Injuries") | brain injur*.ti,kw.  traumatic brain injur*.ti,kw. |
| Respiration, artificial/ | exp artificial ventilation/ | - | mechanical ventilat*.ti,kw. |

**CONCEPT 3: Vitamin D**

| exp Vitamin D/  25 hydroxyvitamin D/  Ergocalciferol/  Cholecalciferol/ | Vitamin D/  25 hydroxyvitamin D/  Ergocalciferol/  Cholecalciferol/ | (MH "Vitamin D+") | Vitamin D.mp.  Calciferols.mp.  Vitamin D2.mp.  Ergocalciferol.mp.  Vitamin D3.mp.  Cholecalciferol.mp.  25-dihydroxycholecalciferol.mp.  Calcitriol.mp. |
| --- | --- | --- | --- |

**Final Search: Concept 1 AND 2 AND 3**

III Material and Methods PRISMA checklist

**PRISMA 2020 for Abstracts Checklist**

| **Section and Topic** | **Item #** | **Checklist item** | **Reported (Yes/No)** |
| --- | --- | --- | --- |
| **TITLE** | | |  |
| Title | 1 | Identify the report as a systematic review. | YES |
| **BACKGROUND** | | |  |
| Objectives | 2 | Provide an explicit statement of the main objective(s) or question(s) the review addresses. | YES |
| **METHODS** | | |  |
| Eligibility criteria | 3 | Specify the inclusion and exclusion criteria for the review. | YES |
| Information sources | 4 | Specify the information sources (e.g. databases, registers) used to identify studies and the date when each was last searched. | YES |
| Risk of bias | 5 | Specify the methods used to assess risk of bias in the included studies. | YES |
| Synthesis of results | 6 | Specify the methods used to present and synthesise results. | YES |
| **RESULTS** | | |  |
| Included studies | 7 | Give the total number of included studies and participants and summarise relevant characteristics of studies. | YES |
| Synthesis of results | 8 | Present results for main outcomes, preferably indicating the number of included studies and participants for each. If meta-analysis was done, report the summary estimate and confidence/credible interval. If comparing groups, indicate the direction of the effect (i.e. which group is favoured). | YES |
| **DISCUSSION** | | |  |
| Limitations of evidence | 9 | Provide a brief summary of the limitations of the evidence included in the review (e.g. study risk of bias, inconsistency and imprecision). | YES |
| Interpretation | 10 | Provide a general interpretation of the results and important implications. | YES |
| **OTHER** | | |  |
| Funding | 11 | Specify the primary source of funding for the review. | NO (other position in the manuscript) |
| Registration | 12 | Provide the register name and registration number. | YES |

*From:*  Page MJ, McKenzie JE, Bossuyt PM, Boutron I, Hoffmann TC, Mulrow CD, et al. The PRISMA 2020 statement: an updated guideline for reporting systematic reviews. BMJ 2021;372:n71. doi: 10.1136/bmj.n71

For more information, visit: <http://www.prisma-statement.org/>

PRISMA 2020 checklist

| **Section and Topic** | **Item #** | **Checklist item** | **Location where item is reported** |
| --- | --- | --- | --- |
| **TITLE** | | |  |
| Title | 1 | Identify the report as a systematic review. | p. 1 |
| **ABSTRACT** | | |  |
| Abstract | 2 | See the PRISMA 2020 for Abstracts checklist. | p. 2 |
| **INTRODUCTION** | | |  |
| Rationale | 3 | Describe the rationale for the review in the context of existing knowledge. | p. 3 |
| Objectives | 4 | Provide an explicit statement of the objective(s) or question(s) the review addresses. | p. 3 |
| **METHODS** | | |  |
| Eligibility criteria | 5 | Specify the inclusion and exclusion criteria for the review and how studies were grouped for the syntheses. | p. 4 |
| Information sources | 6 | Specify all databases, registers, websites, organisations, reference lists and other sources searched or consulted to identify studies. Specify the date when each source was last searched or consulted. | p.4 |
| Search strategy | 7 | Present the full search strategies for all databases, registers and websites, including any filters and limits used. | p.4 +suppl |
| Selection process | 8 | Specify the methods used to decide whether a study met the inclusion criteria of the review, including how many reviewers screened each record and each report retrieved, whether they worked independently, and if applicable, details of automation tools used in the process. | p.4 + p.5 |
| Data collection process | 9 | Specify the methods used to collect data from reports, including how many reviewers collected data from each report, whether they worked independently, any processes for obtaining or confirming data from study investigators, and if applicable, details of automation tools used in the process. | p. 5 |
| Data items | 10a | List and define all outcomes for which data were sought. Specify whether all results that were compatible with each outcome domain in each study were sought (e.g. for all measures, time points, analyses), and if not, the methods used to decide which results to collect. | p. 5 |
|  | 10b | List and define all other variables for which data were sought (e.g. participant and intervention characteristics, funding sources). Describe any assumptions made about any missing or unclear information. | p. 5 + p.6 |
| Study risk of bias assessment | 11 | Specify the methods used to assess risk of bias in the included studies, including details of the tool(s) used, how many reviewers assessed each study and whether they worked independently, and if applicable, details of automation tools used in the process. | p. 5 + p. 6 |
| Effect measures | 12 | Specify for each outcome the effect measure(s) (e.g. risk ratio, mean difference) used in the synthesis or presentation of results. | p. 6 |
| Synthesis methods | 13a | Describe the processes used to decide which studies were eligible for each synthesis (e.g. tabulating the study intervention characteristics and comparing against the planned groups for each synthesis (item #5)). | p. 6 |
|  | 13b | Describe any methods required to prepare the data for presentation or synthesis, such as handling of missing summary statistics, or data conversions. | p. 6 |
|  | 13c | Describe any methods used to tabulate or visually display results of individual studies and syntheses. | p. 6 |
|  | 13d | Describe any methods used to synthesize results and provide a rationale for the choice(s). If meta-analysis was performed, describe the model(s), method(s) to identify the presence and extent of statistical heterogeneity, and software package(s) used. | p. 6 |
|  | 13e | Describe any methods used to explore possible causes of heterogeneity among study results (e.g. subgroup analysis, meta-regression). | p.6 + p.7 |
|  | 13f | Describe any sensitivity analyses conducted to assess robustness of the synthesized results. | p. 6 + p.7 |
| Reporting bias assessment | 14 | Describe any methods used to assess risk of bias due to missing results in a synthesis (arising from reporting biases). | p. 6 |
| Certainty assessment | 15 | Describe any methods used to assess certainty (or confidence) in the body of evidence for an outcome. | p. 7 |
| **RESULTS** | | |  |
| Study selection | 16a | Describe the results of the search and selection process, from the number of records identified in the search to the number of studies included in the review, ideally using a flow diagram. | p.7 +Fig 1 |
|  | 16b | Cite studies that might appear to meet the inclusion criteria, but which were excluded, and explain why they were excluded. | Supl. |
| Study characteristics | 17 | Cite each included study and present its characteristics. | p.7 |
| Risk of bias in studies | 18 | Present assessments of risk of bias for each included study. | Fig 2 |
| Results of individual studies | 19 | For all outcomes, present, for each study: (a) summary statistics for each group (where appropriate) and (b) an effect estimate and its precision (e.g. confidence/credible interval), ideally using structured tables or plots. | Fig2-6 + supl |
| Results of syntheses | 20a | For each synthesis, briefly summarise the characteristics and risk of bias among contributing studies. | Fig 2-6 p.8-9 |
|  | 20b | Present results of all statistical syntheses conducted. If meta-analysis was done, present for each the summary estimate and its precision (e.g. confidence/credible interval) and measures of statistical heterogeneity. If comparing groups, describe the direction of the effect. | Fig 2-6 p.8-9 |
|  | 20c | Present results of all investigations of possible causes of heterogeneity among study results. | Table 1 |
|  | 20d | Present results of all sensitivity analyses conducted to assess the robustness of the synthesized results. | p.9 p10 |
| Reporting biases | 21 | Present assessments of risk of bias due to missing results (arising from reporting biases) for each synthesis assessed. | p. 9 + suppl |
| Certainty of evidence | 22 | Present assessments of certainty (or confidence) in the body of evidence for each outcome assessed. | Fig 2-6 + p. 9-10 |
| **DISCUSSION** | | |  |
| Discussion | 23a | Provide a general interpretation of the results in the context of other evidence. | p. 10-14 |
|  | 23b | Discuss any limitations of the evidence included in the review. | p. 10-14 |
|  | 23c | Discuss any limitations of the review processes used. | p. 10-14 |
|  | 23d | Discuss implications of the results for practice, policy, and future research. | p. 10-14 |
| **OTHER INFORMATION** | | |  |
| Registration and protocol | 24a | Provide registration information for the review, including register name and registration number, or state that the review was not registered. | p. 2 |
|  | 24b | Indicate where the review protocol can be accessed, or state that a protocol was not prepared. | suppl |
|  | 24c | Describe and explain any amendments to information provided at registration or in the protocol. | - |
| Support | 25 | Describe sources of financial or non-financial support for the review, and the role of the funders or sponsors in the review. | p. 16 |
| Competing interests | 26 | Declare any competing interests of review authors. | p. 16 |
| Availability of data, code and other materials | 27 | Report which of the following are publicly available and where they can be found: template data collection forms; data extracted from included studies; data used for all analyses; analytic code; any other materials used in the review. | p. 15 |

*From:*  Page MJ, McKenzie JE, Bossuyt PM, Boutron I, Hoffmann TC, Mulrow CD, et al. The PRISMA 2020 statement: an updated guideline for reporting systematic reviews. BMJ 2021;372:n71. doi: 10.1136/bmj.n71

IV RoB 2

- RoB 2: Overall mortality


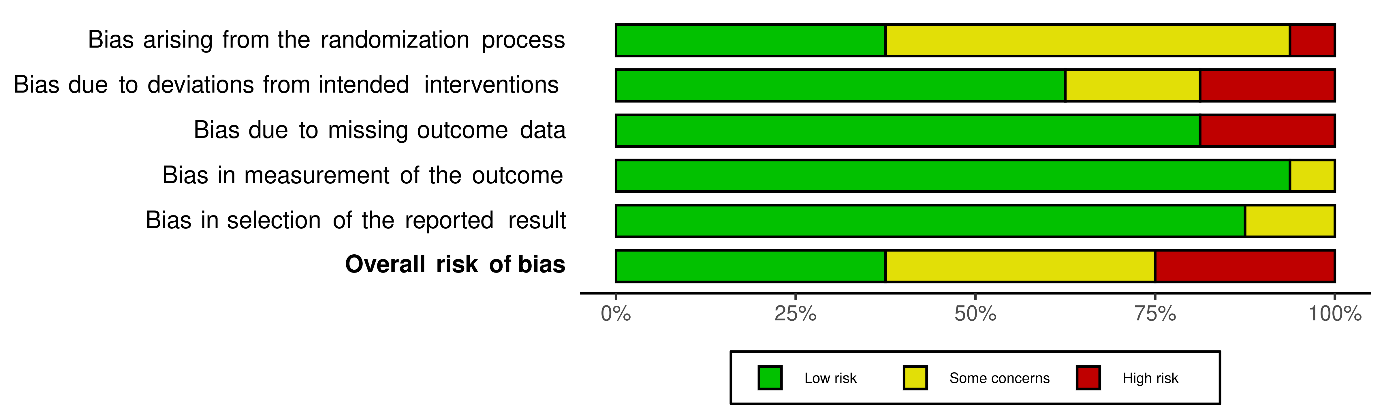


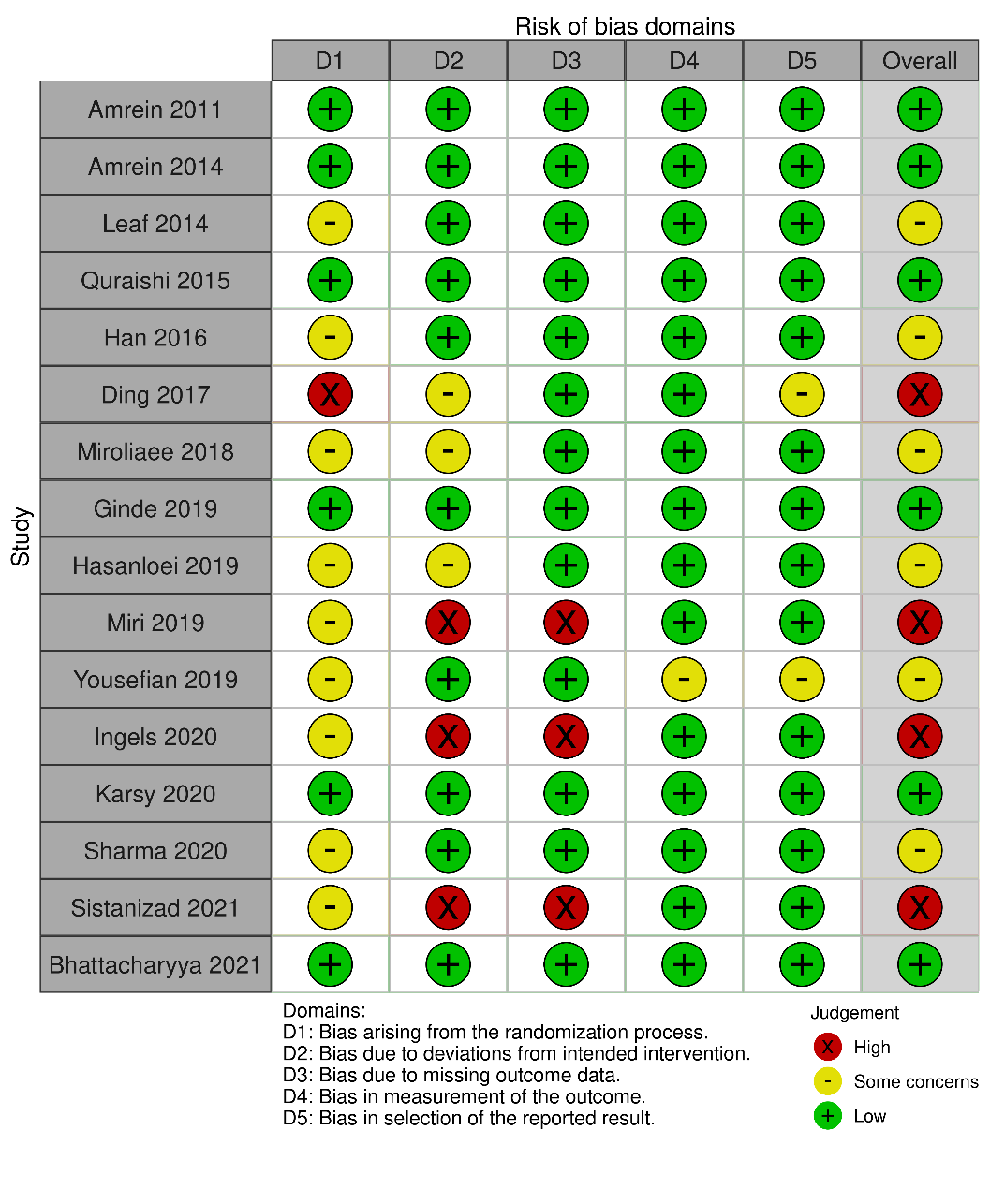


- RoB 2: ICU LOS
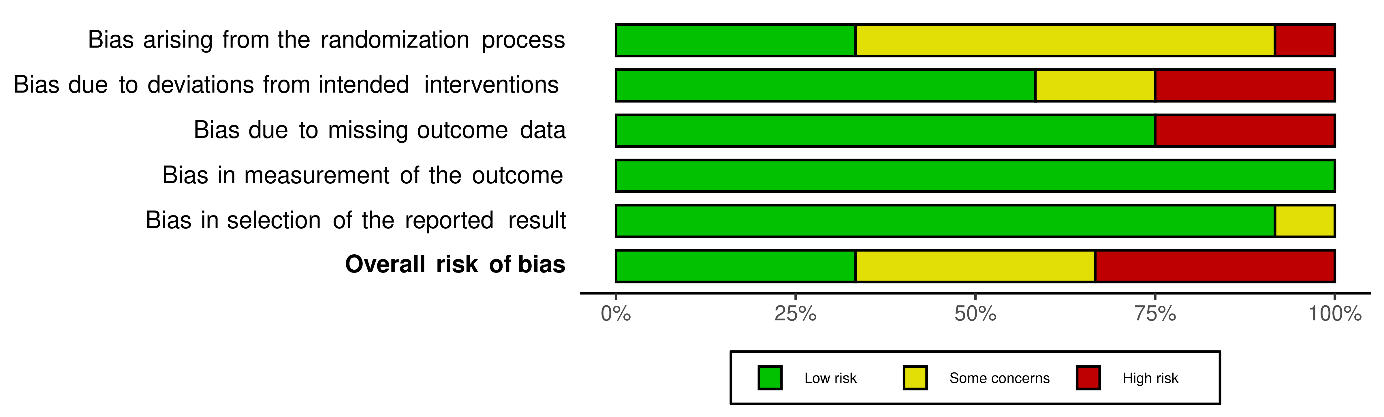

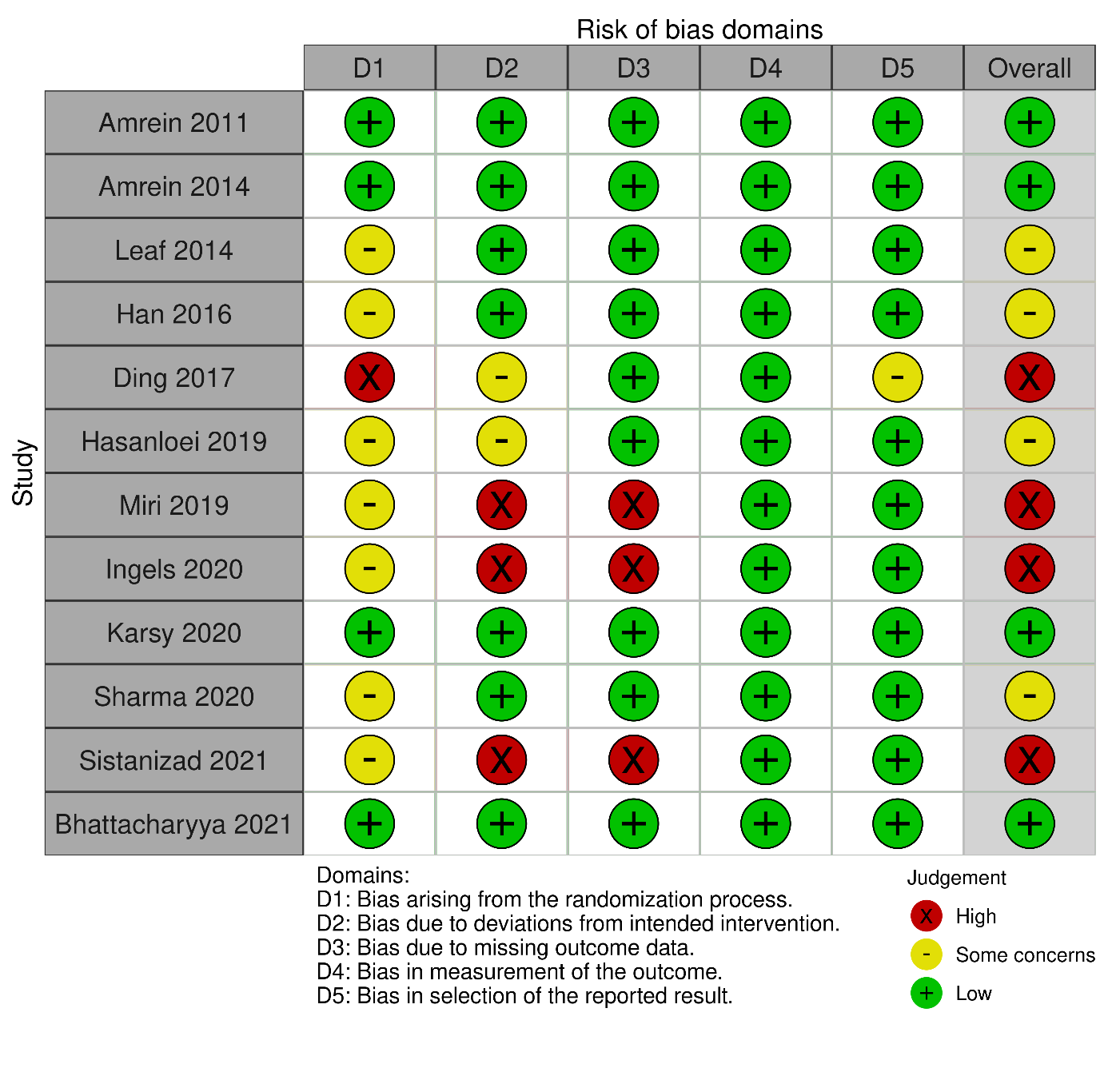

- RoB 2: Hospital LOS
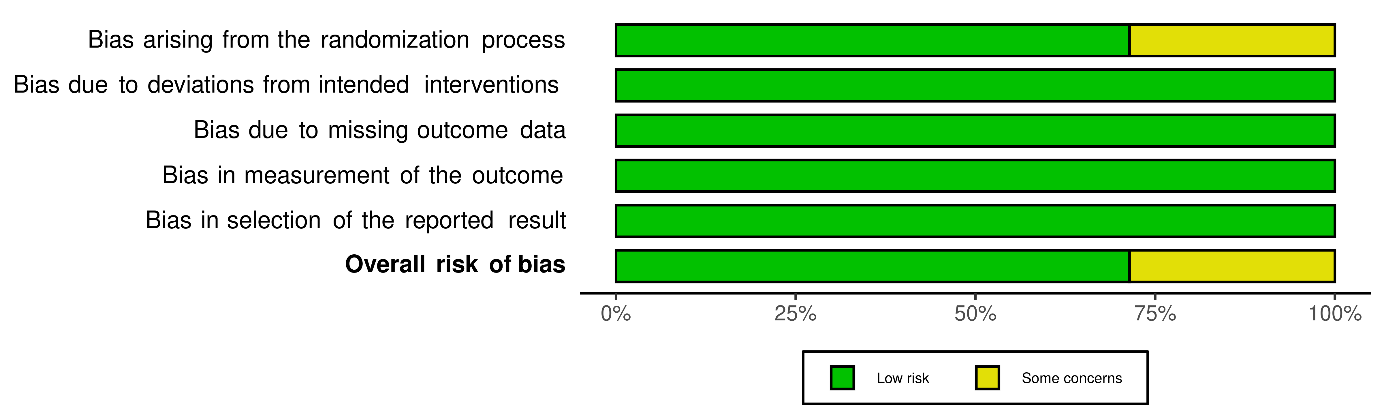

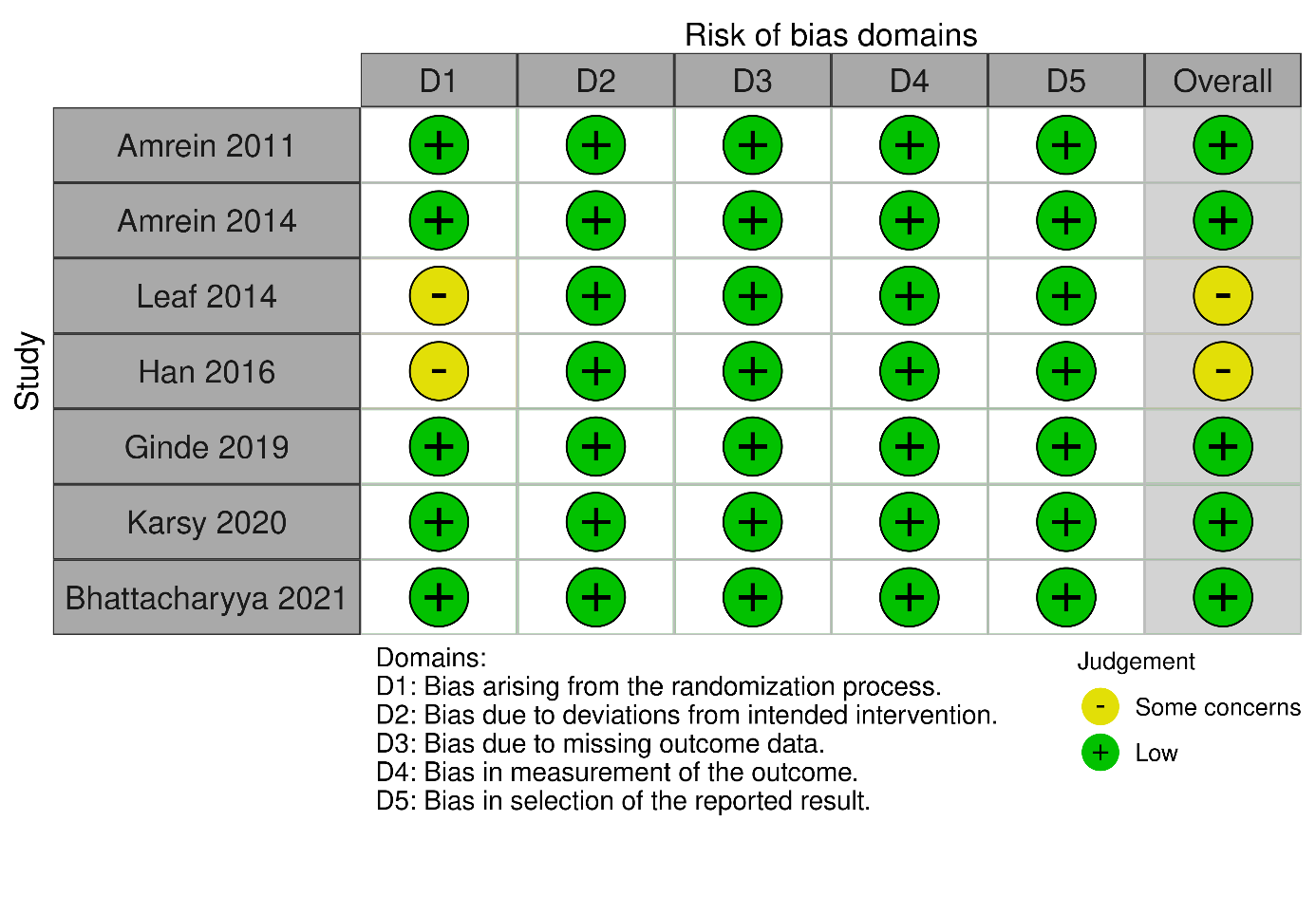

- RoB 2: Duration of mechanical ventilation
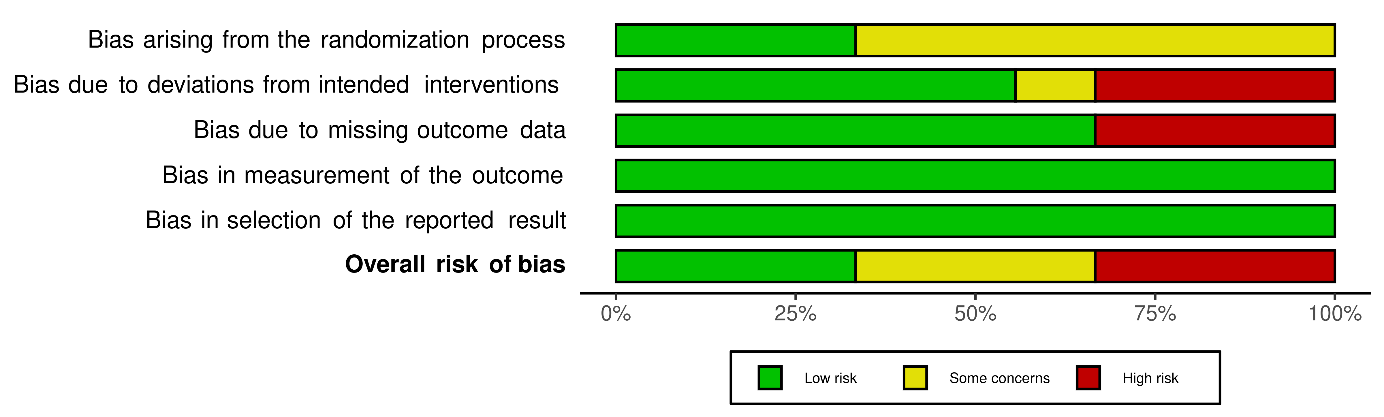

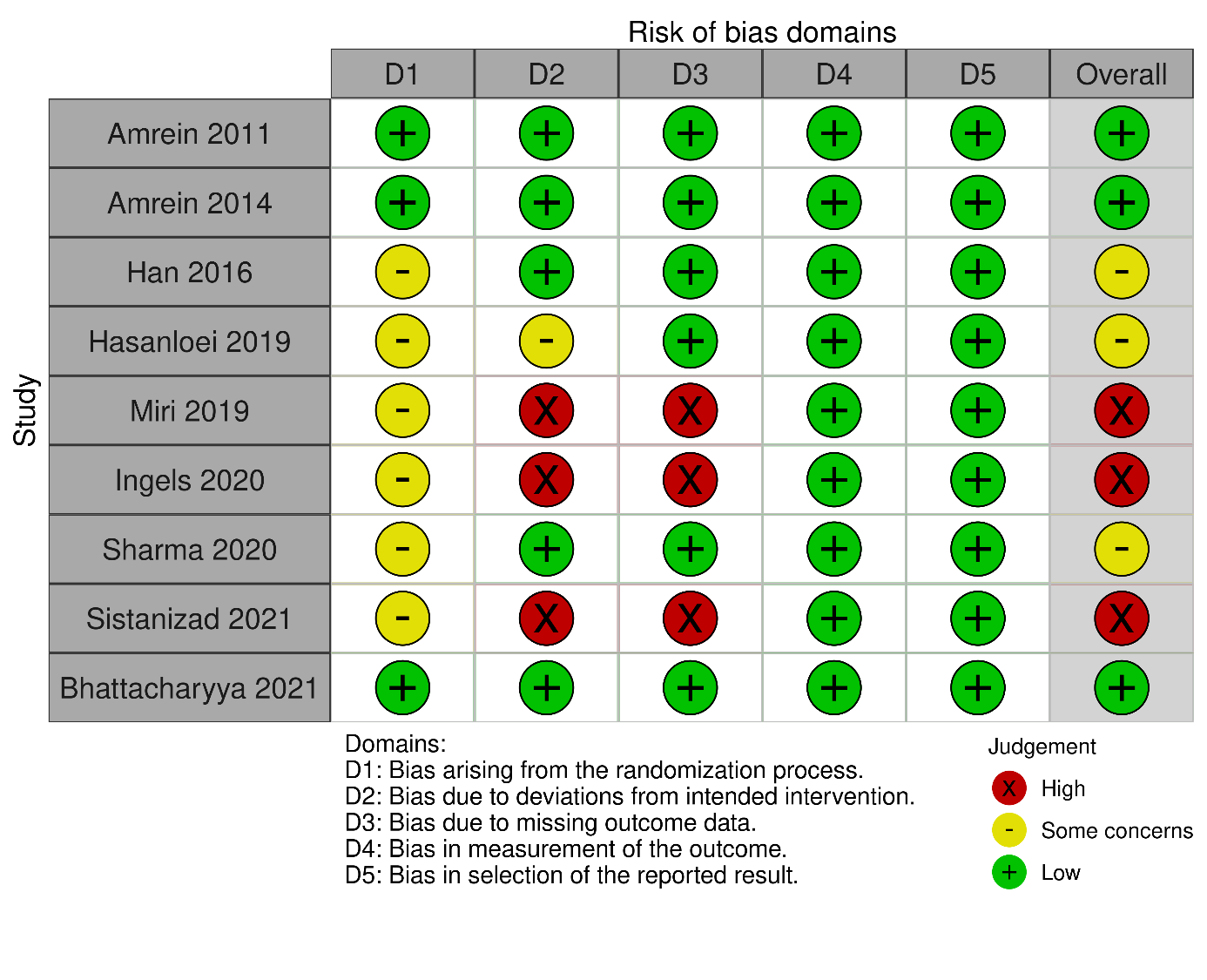


V Trial sequential analysis for overall mortality


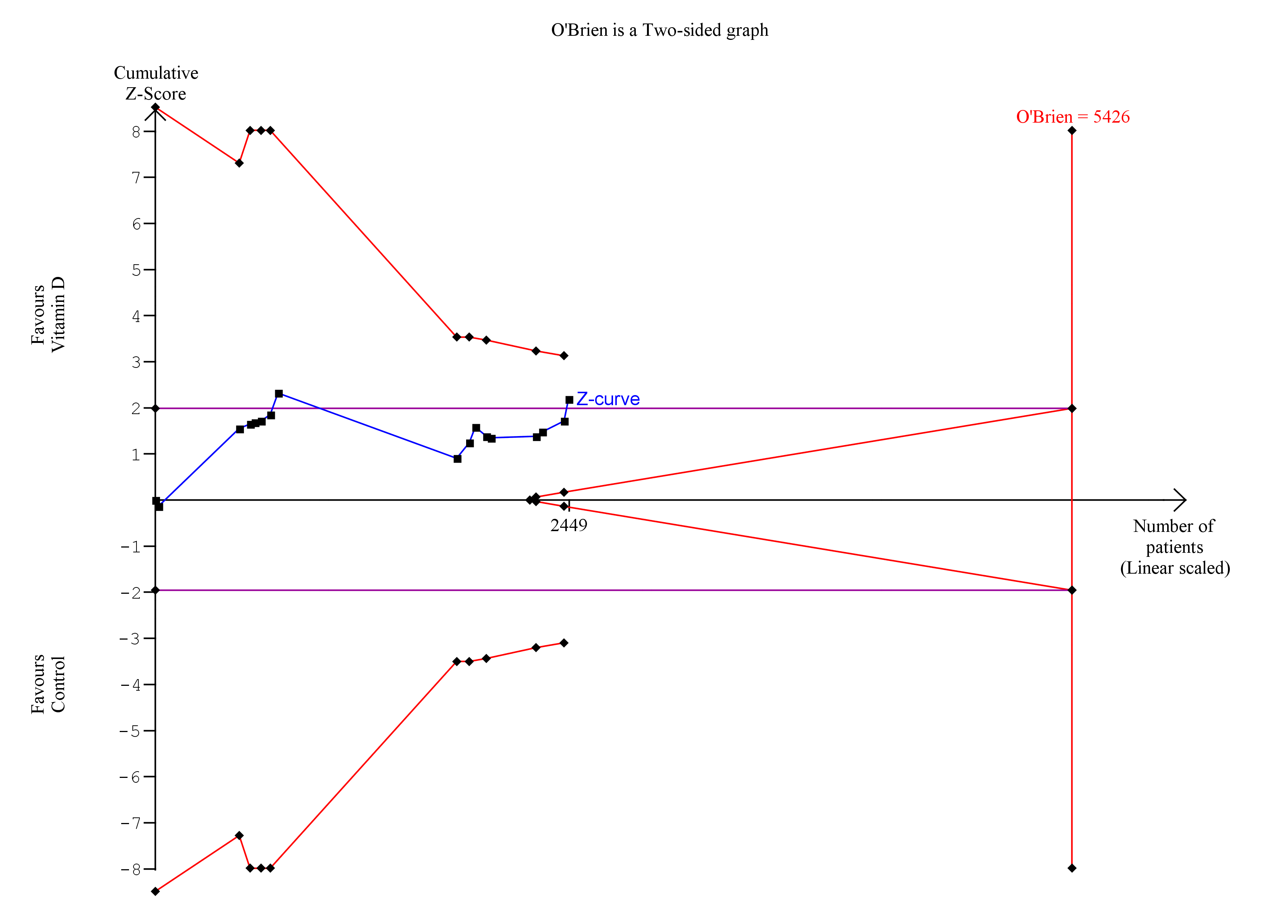


VI Results: Funnel plots

| 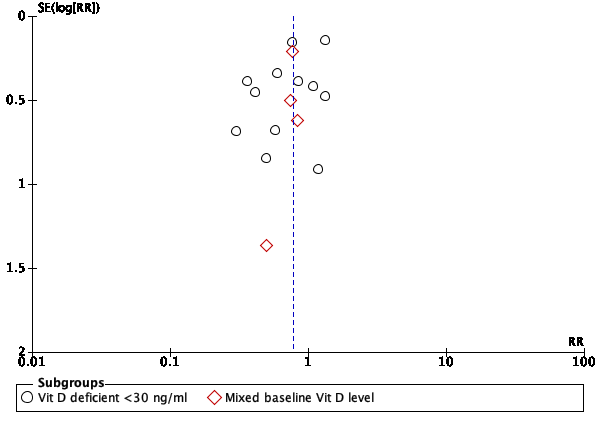  Overall mortality  Eggers' test of the intercept for overall mortality    intercept       95% CI      t     p     -0.735 -1.61 - 0.14 -1.654 0.12  Eggers' test does not indicate the presence of funnel plot asymmetry. |
| --- |
| 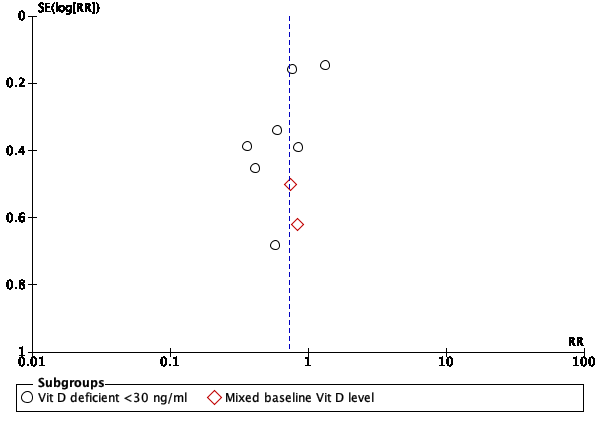  28-d mortality |
| 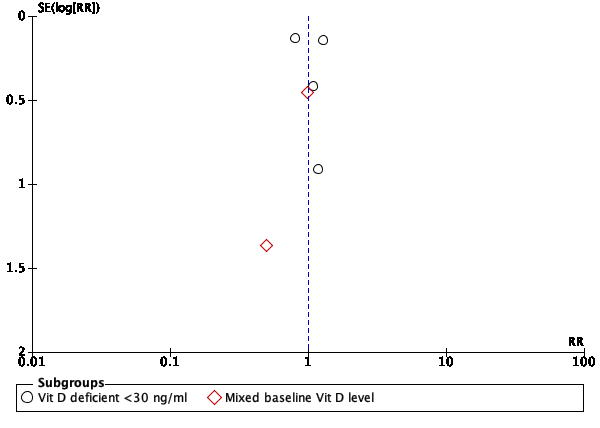  Hospital mortality |
| 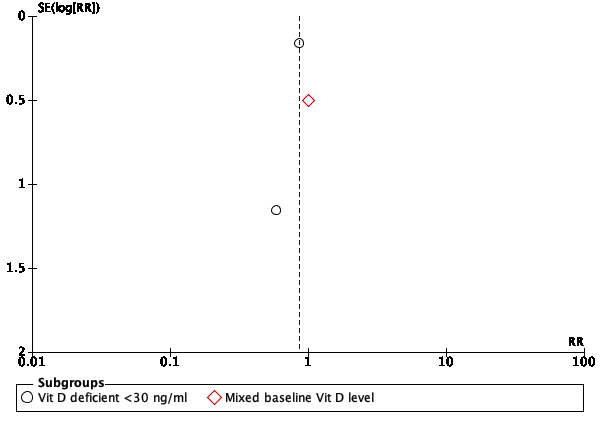  ICU mortality |
| 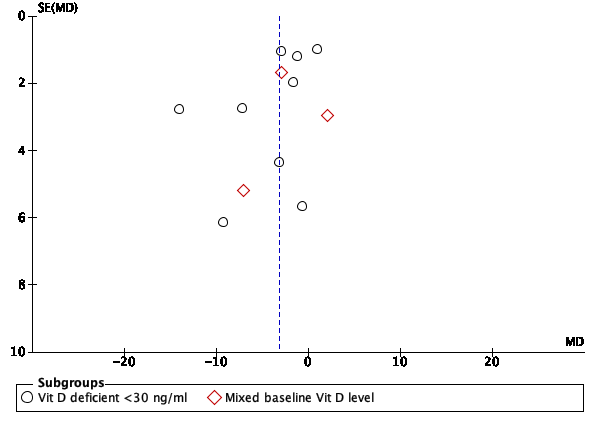  ICU LOS  Eggers' test of the intercept for ICU LOS:  intercept 95% CI t p  -1.562 -3.41 - 0.29 -1.656 0.13  Eggers' test does not indicate the presence of funnel plot asymmetry. |
| 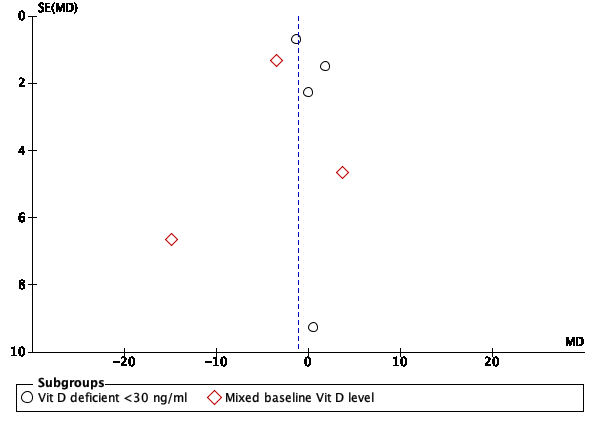  Hospital length of stay |
| 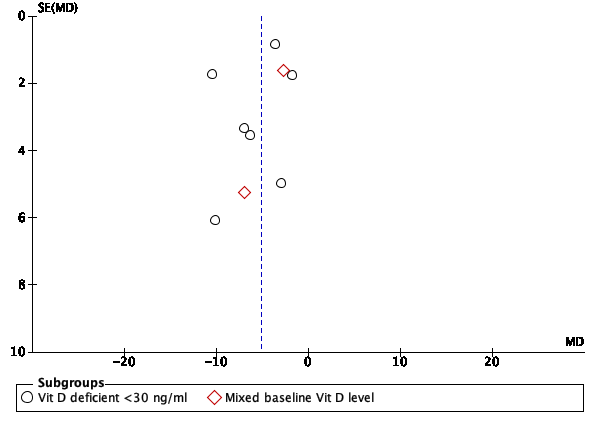  Duration of mechanical ventilation |

Funnel plots of subgroup of EN/PO and IV/IM

|  | EN/PO | IV/IM |
| --- | --- | --- |
| Overall mortality | 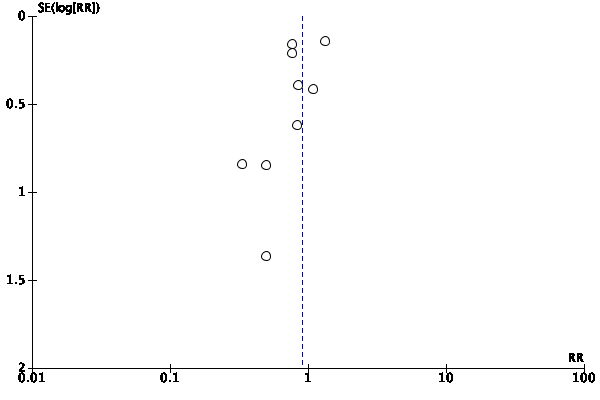 | 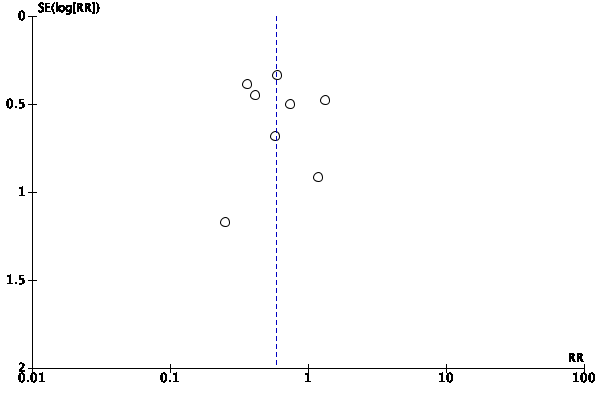 |
| ICU LOS | 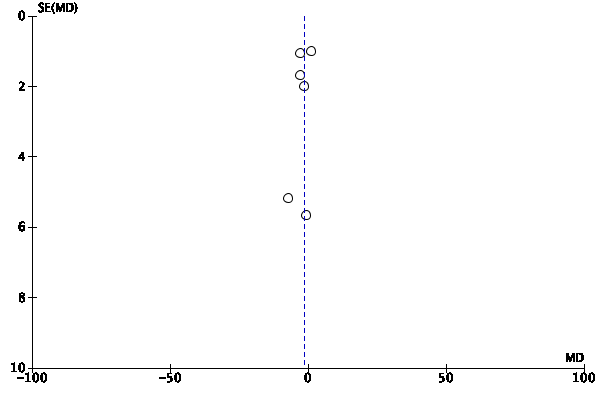 | 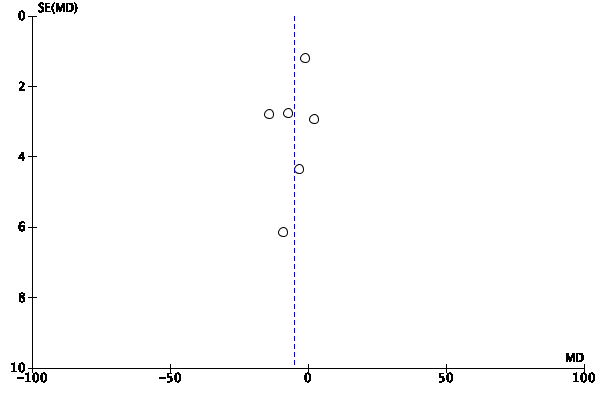 |
| Hosp LOS |  | only 1 study |
| Duration of MV | 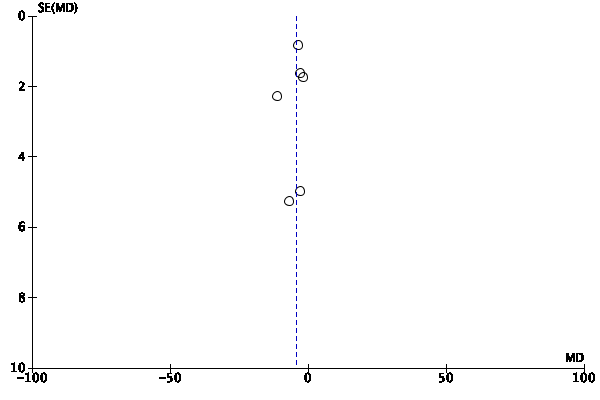 | 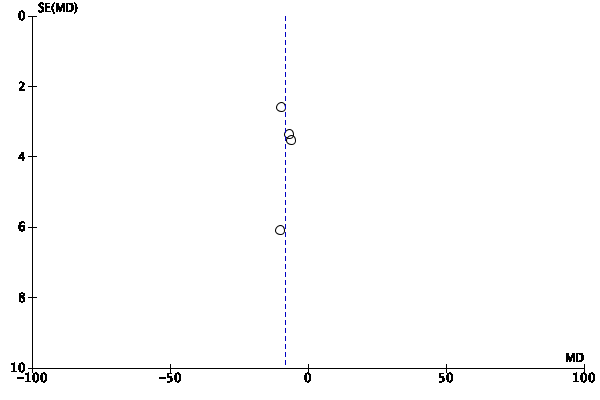 |

VII Results: Excluded studies

**Excluded Studies**

| **No** | **References** | **Reason** |
| --- | --- | --- |
|  | Brock AA, Karsy M, Guan J, Eli I, Menacho ST, Park MS. The Effect of Supplementation of Vitamin D in Neurocritical Care Patients With Hypovitaminosis D: A Randomized Controlled Clinical Trial. Neurosurgery. 2019 Sep 1;66(Supplement_1):nyz310_182. | Abstract of Karsy 2020 |
|  | Aminmansour B, Nikbakht H, Ghorbani A, et al. Comparison of the administration of progesterone versus progesterone and vitamin D in improvement of outcomes in patients with traumatic brain injury: A randomized clinical trial with placebo group. Adv Biomed Res. 2012;1:58. doi:10.4103/2277-9175.100176 | Combined intervention |
|  | Rueda Páez EV, Moncada Parada E, Figueroa Melgarejo J, Ascencio Higuera AA. COMPORTAMIENTO DE CIFRAS DE GLUCEMIA CON LA ADMINISTRACIÓN DE LOS MICRONUTRIENTES VITAMINA D3 Y CALCIO EN PACIENTES NO DIABÉTICOS CON HIPERGLUCEMIA EN UNA UNIDAD DE CUIDADO INTENSIVO DE ADULTOS [Behavior of blood glucose level with the administration of micronutrients vitamin d3 and calcium in nondiabetic patients with hyperglycemia in adult intensive care unit]. Nutr Hosp. 2015;31(5):2103-2108. Published 2015 May 1. doi:10.3305/nh.2015.31.5.8526 | Combined intervention |
|  | Naguib SN, Sabry NA, Farid SF, Alansary AM. Short-term Effects of Alfacalcidol on Hospital Length of Stay in Patients Undergoing Valve Replacement Surgery: A Randomized Clinical Trial. Clin Ther. 2021;43(1):e1-e18. doi:10.1016/j.clinthera.2020.11.008 | Elective surgery patients |
|  | Parekh D, Dancer RCA, Scott A, et al. Vitamin D to Prevent Lung Injury Following Esophagectomy-A Randomized, Placebo-Controlled Trial. Crit Care Med. 2018;46(12):e1128-e1135. doi:10.1097/CCM.0000000000003405 | Elective surgery patients |
|  | Han JH, Ginde AA, Brown SM, et al. Effect of Early High-Dose Vitamin D3 Repletion on Cognitive Outcomes in Critically Ill Adults. Chest. 2021;160(3):909-918. doi:10.1016/j.chest.2021.03.046 | Follow up of Ginde 2019 |
|  | Smith EM, Jones JL, Han JE, et al. High-Dose Vitamin D3 Administration Is Associated With Increases in Hemoglobin Concentrations in Mechanically Ventilated Critically Ill Adults: A Pilot Double-Blind, Randomized, Placebo-Controlled Trial. JPEN J Parenter Enteral Nutr. 2018;42(1):87-94. doi:10.1177/0148607116678197 | No clinical outcome |
|  | Alizadeh N, Khalili H, Mohammadi M, Abdollahi A, Ala S. Effect of vitamin D on stress-induced hyperglycaemia and insulin resistance in critically ill patients. Int J Clin Pract. 2016;70(5):396-405. doi:10.1111/ijcp.12795 | No clinical outcomes. 3 deaths not specified to group. |
|  | Nair P, Venkatesh B, Lee P, et al. A Randomized Study of a Single Dose of Intramuscular Cholecalciferol in Critically Ill Adults. Crit Care Med. 2015;43(11):2313-2320. doi:10.1097/CCM.0000000000001201 | No control group (higher vs lower dose of Vit D) |
|  | Padhy SS, Malviya D, Harjai M, Tripathi M, Das PK, Rastogi S. A Study of Vitamin D Level in Critically Ill Patients and Effect of Supplementation on Clinical Outcome. Anesth Essays Res. 2020;14(3):474-477. doi:10.4103/aer.AER_83_20 | No control group (higher vs lower dose of Vit D) |
|  | Grossmann RE, Zughaier SM, Kumari M, et al. Pilot study of vitamin D supplementation in adults with cystic fibrosis pulmonary exacerbation: A randomized, controlled trial. Dermatoendocrinol. 2012;4(2):191-197. doi:10.4161/derm.20332 | Not critically ill patients |
|  | Jokar A, Ahmadi K, Taherinia A, Didgar F, Kazemi F, Bahramian M. The Effects of Injected Vitamin D on Prognosis of Patients with Urosepsis. Horm Metab Res. 2018;50(5):383-388. doi:10.1055/a-0595-7731 | Not critically ill patients |
|  | Maghbooli Z, Sahraian MA, Jamali-Moghadam SR, et al. Treatment with 25-hydroxyvitamin D3 (calcifediol) is associated with a reduction in the blood neutrophil-to-lymphocyte ratio marker of disease severity in patients hospitalized with COVID-19: a pilot, multicenter, randomized, placebo-controlled double blind clinical trial [published online ahead of print, 2021 Oct 12]. Endocr Pract. 2021;S1530-891X(21)01259-3. doi:10.1016/j.eprac.2021.09.016 | Not critically ill patients |
|  | Murai IH, Fernandes AL, Sales LP, et al. Effect of a Single High Dose of Vitamin D3 on Hospital Length of Stay in Patients With Moderate to Severe COVID-19: A Randomized Clinical Trial. JAMA. 2021;325(11):1053-1060. doi:10.1001/jama.2020.26848 | Not critically ill patients |
|  | Rousseau AF, Foidart-Desalle M, Ledoux D, et al. Effects of cholecalciferol supplementation and optimized calcium intakes on vitamin D status, muscle strength and bone health: a one-year pilot randomized controlled trial in adults with severe burns. Burns. 2015;41(2):317-325. doi:10.1016/j.burns.2014.07.005 | Not critically ill patients |
|  | Slow S, Epton M, Storer M, et al. Effect of adjunctive single high-dose vitamin D3 on outcome of community-acquired pneumonia in hospitalised adults: The VIDCAPS randomised controlled trial. Sci Rep. 2018;8(1):13829. Published 2018 Sep 14. doi:10.1038/s41598-018-32162-2 | Not critically ill patients |
|  | Abbasi S, Alikiaii B, Kashefi P, Hatamzadeh B. The Effect of Vitamin D Supplement on Outcomes in Patients with Traumatic Injuries Hospitalized in Intensive Care Unit: A Clinical Trial Study. J Isfahan Med Sch 2020; 38(590): 649-54. | Not RCT |
|  | Quraishi SA, Bhan I, Matthay MA, Thompson BT, Camargo CA Jr, Bajwa EK. Vitamin D Status and Clinical Outcomes in Acute Respiratory Distress Syndrome: A Secondary Analysis From the Assessment of Low Tidal Volume and Elevated End-Expiratory Volume to Obviate Lung Injury (ALVEOLI) Trial [published online ahead of print, 2021 Jun 24]. J Intensive Care Med. 2021;8850666211028139. doi:10.1177/08850666211028139 | Secondary analysis of Quraishi 2015 |
|  | Arabi SM, Bahrami LS, Ranjbar G, Tabesh H, Norouzy A. The effect of vitamin D supplementation on inflammation in critically ill patients: A systematic review. PharmaNutrition. 2020 Sep 1;13:100196. | Systematic review |
|  | Lan SH, Lai CC, Chang SP, Lu LC, Hung SH, Lin WT. Vitamin D supplementation and the outcomes of critically ill adult patients: a systematic review and meta-analysis of randomized controlled trials. Sci Rep. 2020;10(1):14261. Published 2020 Aug 31. doi:10.1038/s41598-020-71271-9 | Systematic review |
|  | Langlois PL, Szwec C, D'Aragon F, Heyland DK, Manzanares W. Vitamin D supplementation in the critically ill: A systematic review and meta-analysis. Clin Nutr. 2018;37(4):1238-1246. doi:10.1016/j.clnu.2017.05.006 | Systematic review |
|  | Peng L, Li L, Wang P, et al. Association between Vitamin D supplementation and mortality in critically ill patients: A systematic review and meta-analysis of randomized clinical trials. PLoS One. 2020;15(12):e0243768. Published 2020 Dec 14. doi:10.1371/journal.pone.0243768 | Systematic review |
|  | Putzu A, Belletti A, Cassina T, et al. Vitamin D and outcomes in adult critically ill patients. A systematic review and meta-analysis of randomized trials. J Crit Care. 2017;38:109-114. doi:10.1016/j.jcrc.2016.10.029 | Systematic review |
|  | Shen H, Mei Y, Zhang K, Xu X. The Effect of Vitamin D Supplementation on Clinical Outcomes for Critically Ill Patients: A Systemic Review and Meta-Analysis of Randomized Clinical Trials. Front Nutr. 2021;8:664940. Published 2021 May 4. doi:10.3389/fnut.2021.664940 | Systematic review |
|  | Weng H, Li JG, Mao Z, Zeng XT. Randomised trials of vitamin D3 for critically ill patients in adults: systematic review and meta-analysis with trial sequential analysis. Intensive Care Med. 2017;43(2):277-278. doi:10.1007/s00134-016-4591-1 | Systematic review |
|  | Yang C, Lu Y, Wan M, et al. Efficacy of High-Dose Vitamin D Supplementation as an Adjuvant Treatment on Pneumonia: Systematic Review and a Meta-Analysis of Randomized Controlled Studies. Nutr Clin Pract. 2021;36(2):368-384. doi:10.1002/ncp.10585 | Systematic review |
|  | Maghbooli Z, Sahraian MA, Jamalimoghadamsiahkali S, et al. Treatment With 25-Hydroxyvitamin D3 (Calcifediol) Is Associated With a Reduction in the Blood Neutrophil-to-Lymphocyte Ratio Marker of Disease Severity in Hospitalized Patients With COVID-19: A Pilot Multicenter, Randomized, Placebo-Controlled, Double-Blinded Clinical Trial. Endocr Pract. 2021;. doi:10.1016/j.eprac.2021.09.016, 10.1016/j.eprac.2021.09.016 | Not critically ill patients |
|  | Murai IH, Fernandes AL, Sales LP, Pinto AJ, Goessler KF, Duran CSC, Silva CBR, Franco AS, Macedo MB, Dalmolin HHH, Baggio J, Balbi GGM, Reis BZ, Antonangelo L, Caparbo VF, Gualano B, Pereira RMR, et al. Effect of a Single High Dose of Vitamin D3 on Hospital Length of Stay in Patients With Moderate to Severe COVID-19: a Randomized Clinical Trial JAMA. 2021;2021;2021;2021;2021;2021;2021;2021; Available from EBM Reviews - Cochrane Central Register of Controlled Trials at http://ovidsp.ovid.com/ovidweb.cgi?T=JS&PAGE=reference&D=cctr&NEWS=N&AN=CN-02252719. Accessed November 12, 2021. | Not critically ill patients |

VIII Registered ongoing studies

**Ongoing Studies from ClinicalTrials.gov**

| **No** | **Registration Number** | **Country** | **Title** | **Acronym** | **N** |
| --- | --- | --- | --- | --- | --- |
| 1 | NCT04292873 | Taiwan | Effects of Enteral Supplement Vitamin D Incritically Ill Patients | - | 60 |
| 2 | NCT05094388 | Austria | The VITDALIZE Study: Effect of High-dose Vitamin D3 on 28-day Mortality in Adult Critically Ill Patients (VITDALIZE) | VITDALIZE | 2400 |

Using keyword 'critical illness' and 'Vitamin D' – 2 active studies found in ClinicalTrials.gov

IX Results: Subgroup single vs multicentre study

Overall mortality: single vs multicenter


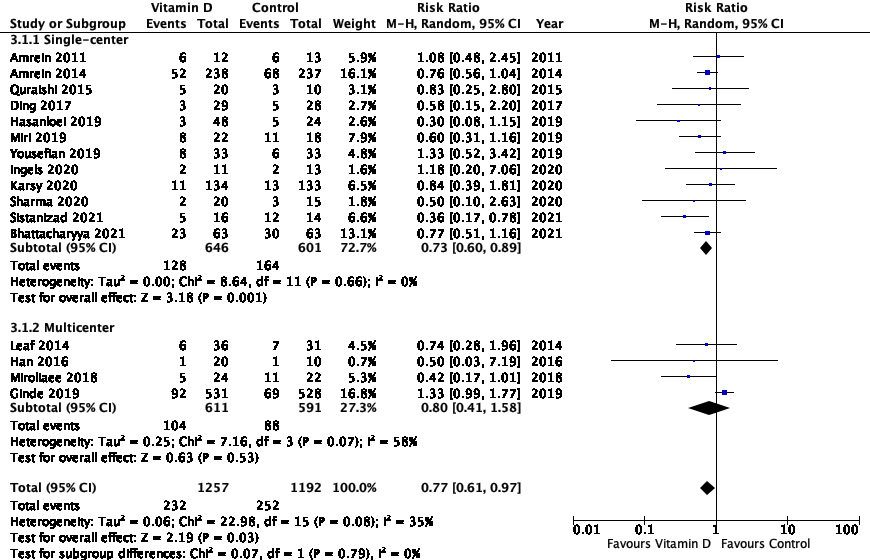


28-day mortality: single vs multicenter


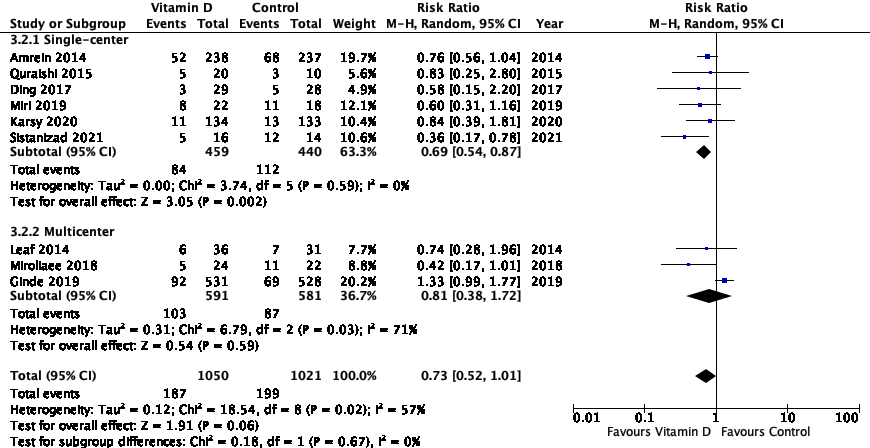


ICU LOS: single vs multicenter


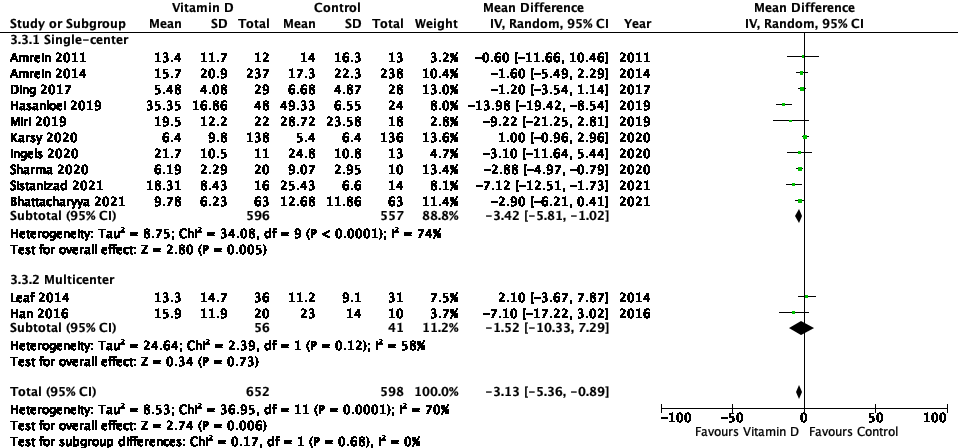


Duration of MV: single vs multicenter


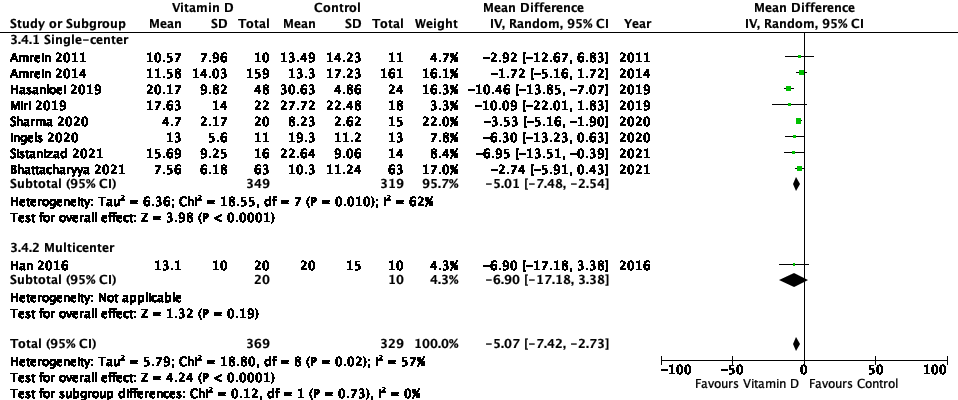


X Results: GRADE evidence profile

| **Certainty assessment** | | | | | | | **№ of patients** | | **Effect** | | **Certainty** | **Importance** |
| --- | --- | --- | --- | --- | --- | --- | --- | --- | --- | --- | --- | --- |
| **№ of studies** | **Study design** | **Risk of bias** | **Inconsistency** | **Indirectness** | **Imprecision** | **Other considerations** | **Vit D** | **Placebo (EN/PO** | **Relative (95% CI)** | **Absolute (95% CI)** |  |  |
| **Overall Mortality** | | | | | | | | | | | | |
| 16 | randomised trials | serious^a^ | not serious^b^ | not serious | serious^c^ | none | 232/1257 (18.5%) | 252/1192 (21.1%) | **RR 0.78** (0.62 to 0.97) | **47 fewer per 1.000** (from 80 fewer to 6 fewer) | ⨁⨁◯◯ Low | CRITICAL |
| **ICU LOS** | | | | | | | | | | | | |
| 12 | randomised trials | serious^d^ | serious^e^ | not serious | not serious^f^ | none | 652 | 598 | - | MD **3.13 lower** (5.36 lower to 0.89 lower) | ⨁⨁◯◯ Low | IMPORTANT |
| **Hosp LOS** | | | | | | | | | | | | |
| 7 | randomised trials | not serious | serious^g^ | not serious | serious^h^ | none | 912 | 909 | - | MD **1.04 lower** (3.23 lower to 1.15 higher) | ⨁⨁◯◯ Low | IMPORTANT |
| **Mechanical Ventilation** | | | | | | | | | | | | |
| 9 | randomised trials | serious^i^ | serious^j^ | not serious | not serious^k^ | none | 369 | 329 | - | MD **5.47 lower** (7.69 lower to 3.24 lower) | ⨁⨁◯◯ Low | IMPORTANT |

**CI:** confidence interval; **MD:** mean difference; **RR:** risk ratio

**Explanations**

a. A quarter (4/16) of all studies have a high risk of bias according to Cochrane Risk of Bias 2 assessment. Those four studies represent 6.2% (151/2449) of the study population and a weight of 18.3% in the meta-analysis.

b. The biggest study included (Ginde 2019) with a low risk of bias according to Cochrane Risk of Bias 2 assessment reports no benefit of Vitamin D compared to placebo and stands in contrast to many smaller studies. Nevertheless I² was low (30%), heterogeneity was non-significant (p=0.12) and ~90% of the confidence intervals are overlapping. In this borderline decision we decided not to downgrade certainty.

c. Inconclusive TSA and unmet information size.

d. A third (4/12) of all studies have a high risk of bias according to Cochrane Risk of Bias 2 assessment. Those four studies represent 12.1% (151/1250) of the study population and a weight of 28.6% in the meta-analysis.

e. Heterogeneity (p<0.001), I²=70% and non-overlapping confidence intervals lead to a downgrade.

f. Wide confidence interval would be a reason to downgrade because of imprecision but even the upper boundary of 0.89 days less on intensive care unit seem clinical relevant. Therefore no downgrade was applied.

g. Moderate I² (54%), overlapping confidence intervals and significant heterogeneity (p= 0.05) lead to a downgrade

h. As confidence interval crosses 0 no clear recommendation can be given by these results

i. A third (3/9) of all studies have a high risk of bias according to Cochrane Risk of Bias 2 assessment. Those three studies represent 13.5% (94/698) of the study population and a weight of 17.8% in the meta-analysis.

j. Moderate I² (54%), significant heterogeneity (p=0.02) and ~10% of studies confidence intervals are not overlapping

k. Wide confidence interval would be reason to downgrade because of imprecision but even the upper boundary of 3.24 days less on mechanical ventilation seem clinical relevant. Therefore no downgrade was applied
