# Supplementary figures and images for: Administration of vitamin D and its metabolites in critically ill adult patients: an updated systematic review with meta-analysis of randomized controlled trials
Source: Crit Care. 2022 Sep 6;26:268. doi: 10.1186/s13054-022-04139-1 (PMC9446655; doi:10.1186/s13054-022-04139-1)

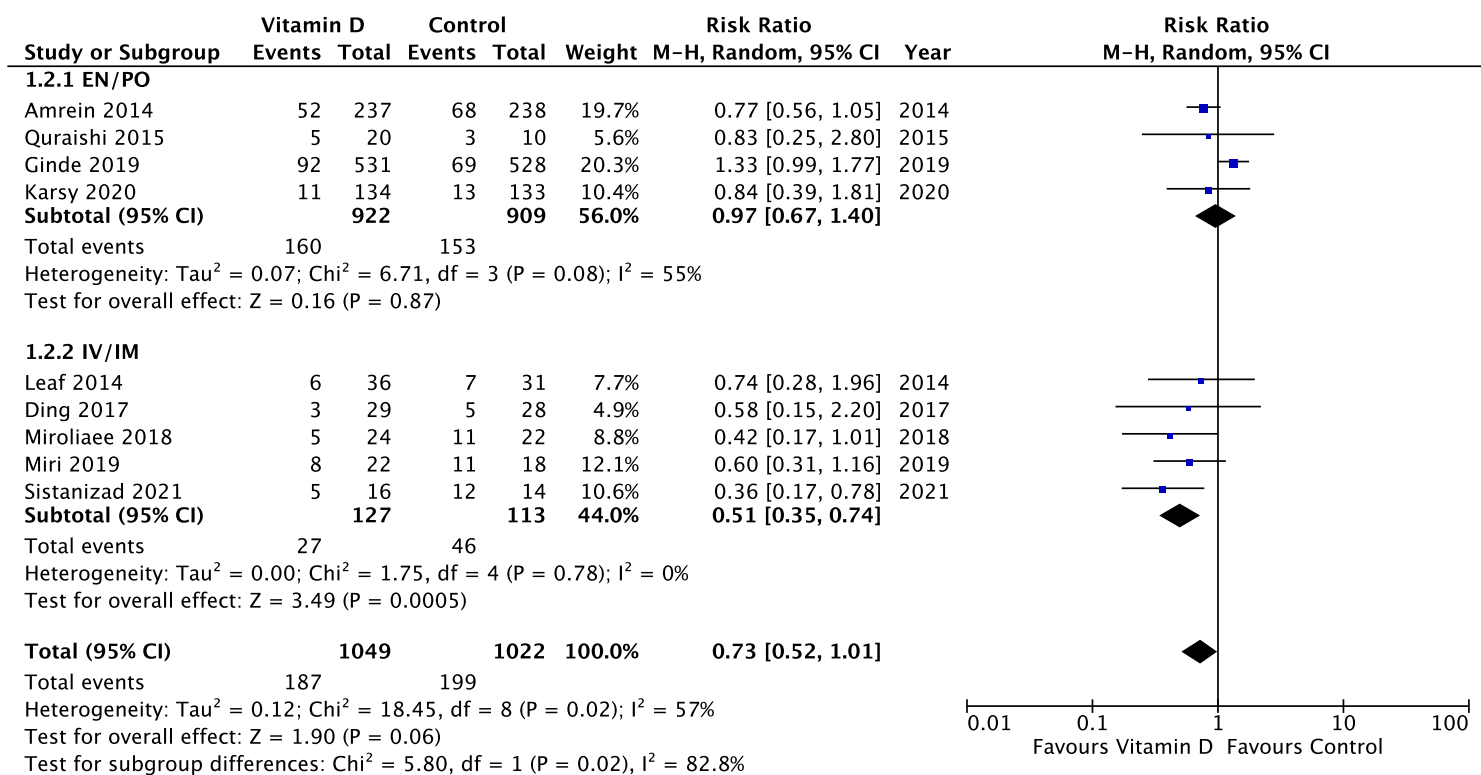

Supplement: Supplementary file 2 — Additional file 2. Mortality 28-day in critically ill patients: Vitamin D compared to placebo (or standard of care) including subgroup analysis of route of administration. [file 13054_2022_4139_MOESM2_ESM.pdf]

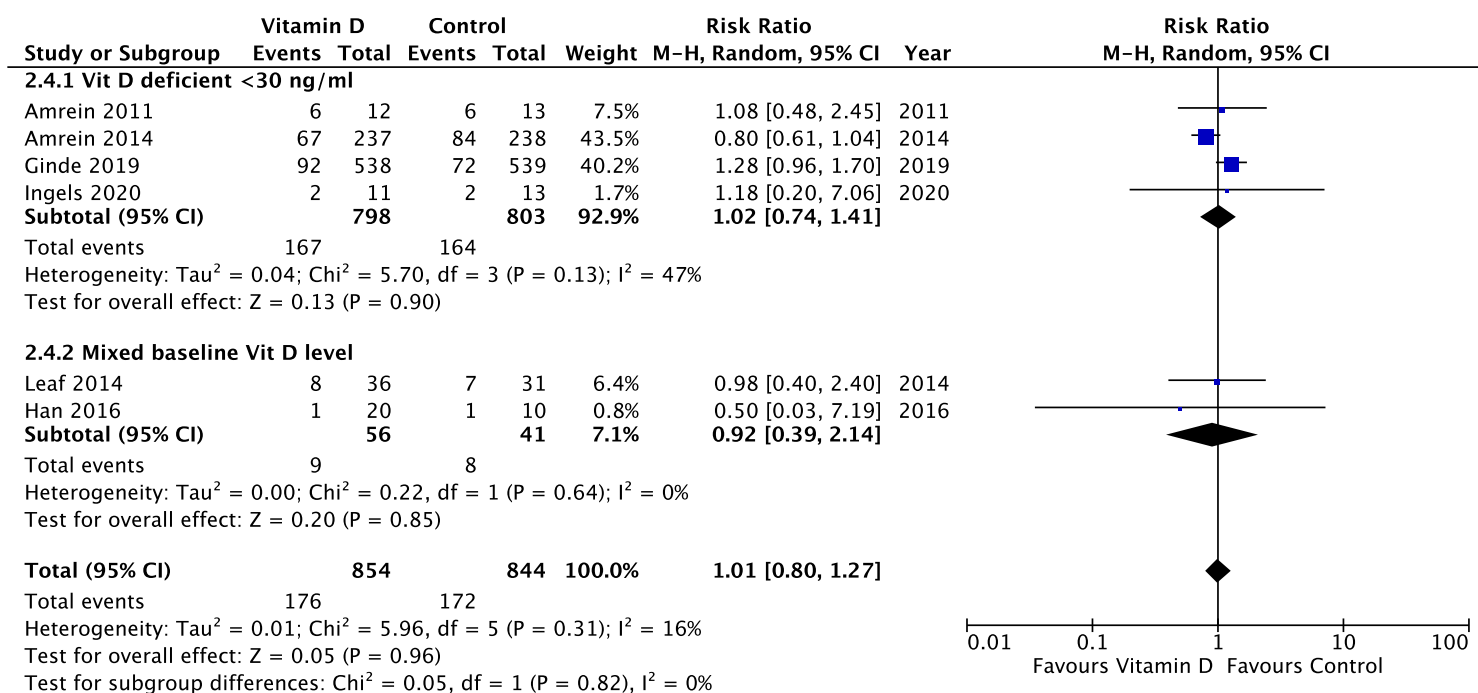

Supplement: Supplementary file 3 — Additional file 3. Hospital mortality: Vitamin D compared to placebo or standard of care for critically ill patients including subgroup analysis of baseline vitamin D. [file 13054_2022_4139_MOESM3_ESM.pdf]

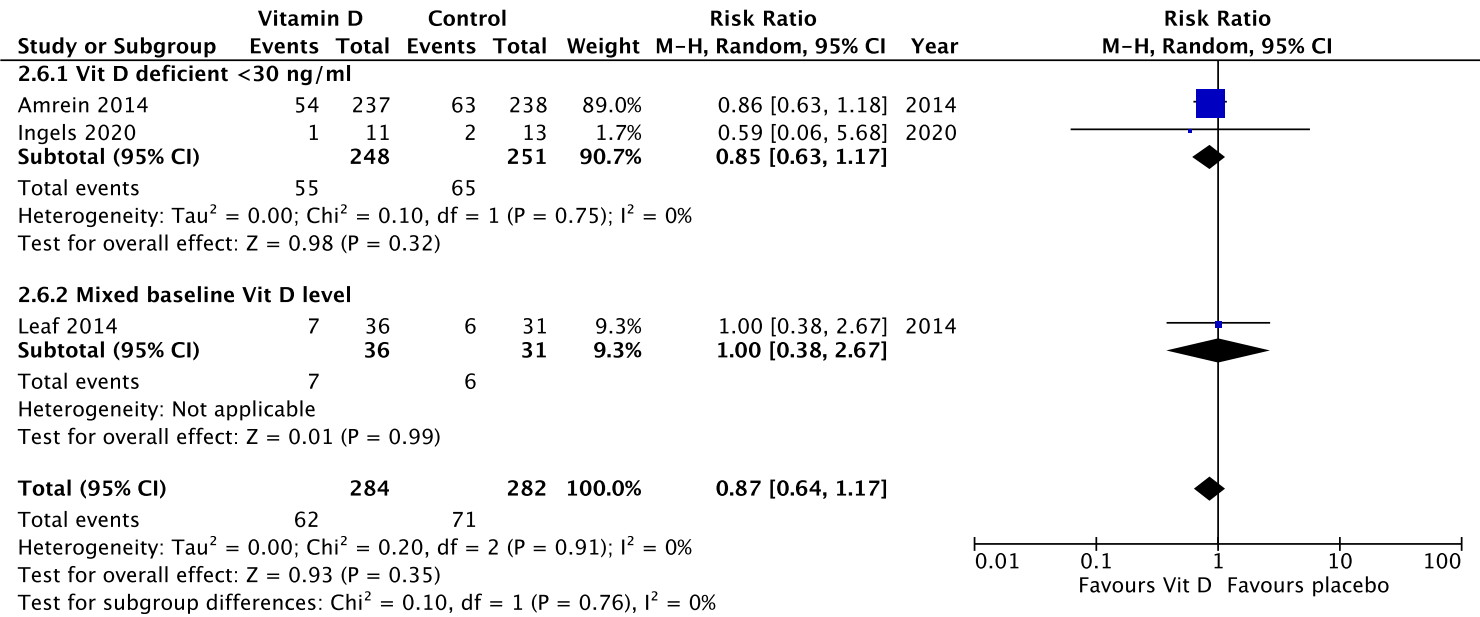

Supplement: Supplementary file 4 — Additional file 4. ICU mortality: Vitamin D compared to placebo or standard of care for critically ill patients including subgroup analysis of baseline vitamin D. [file 13054_2022_4139_MOESM4_ESM.pdf]

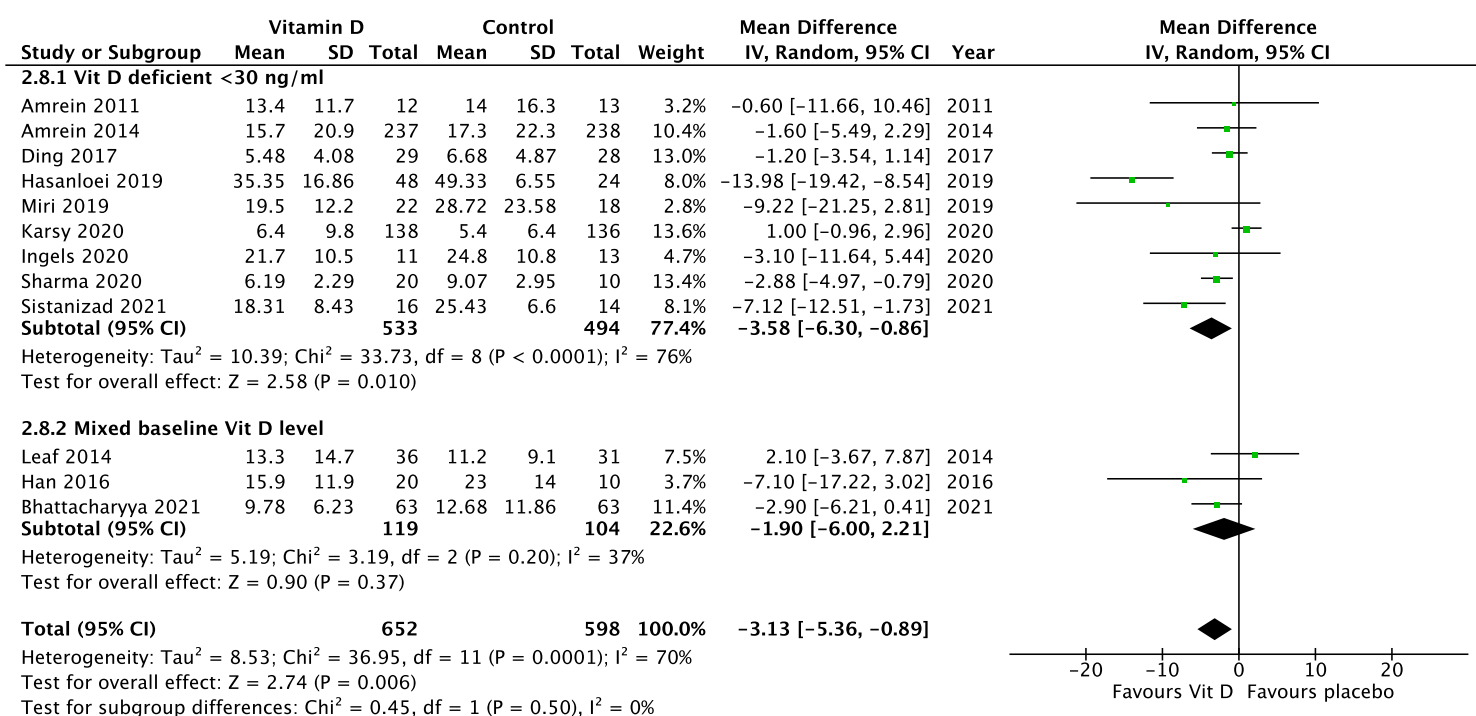

Supplement: Supplementary file 5 — Additional file 5. ICU length of stay: Vitamin D compared to placebo or standard of care for critically ill patients including subgroup analysis of baseline vitamin D. [file 13054_2022_4139_MOESM5_ESM.pdf]

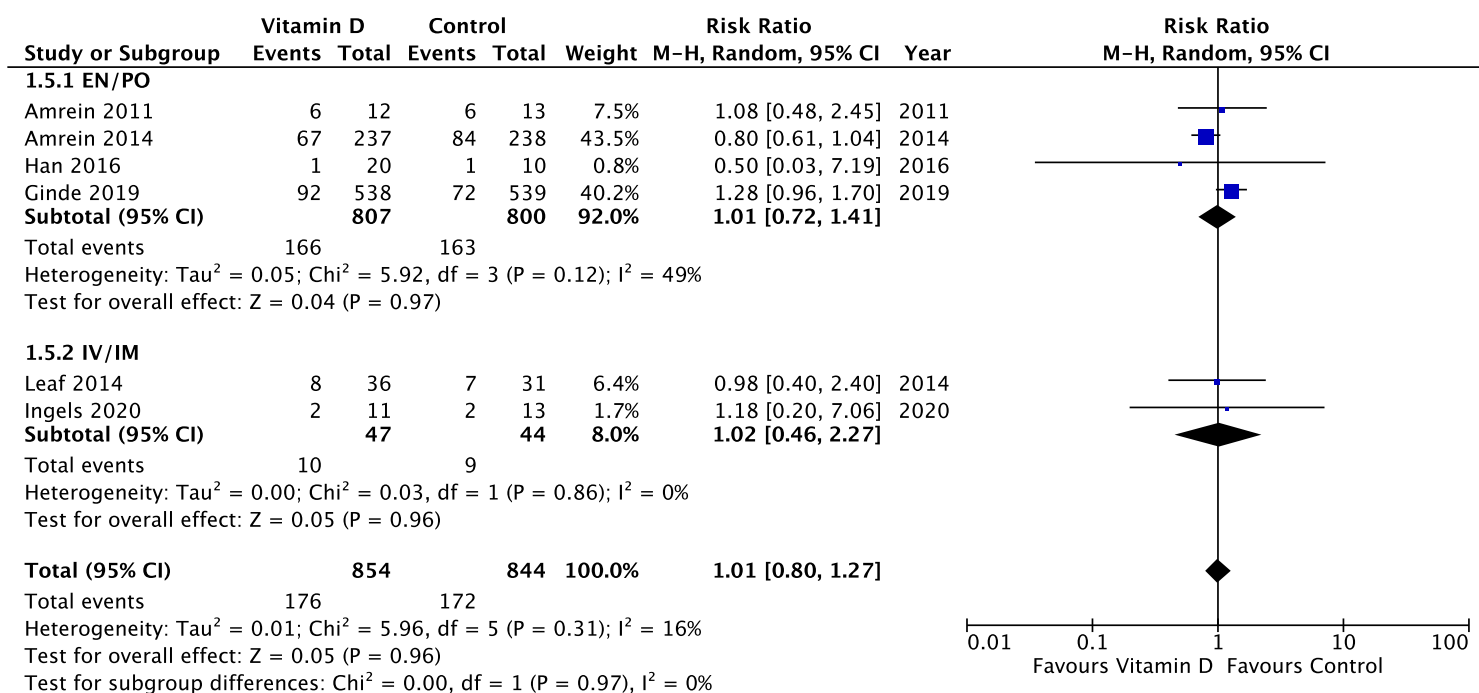

Supplement: Supplementary file 6 — Additional file 6. Hospital mortality: Vitamin D compared to placebo or standard of care for critically ill patients including subgroup analysis of route of administration. [file 13054_2022_4139_MOESM6_ESM.pdf]

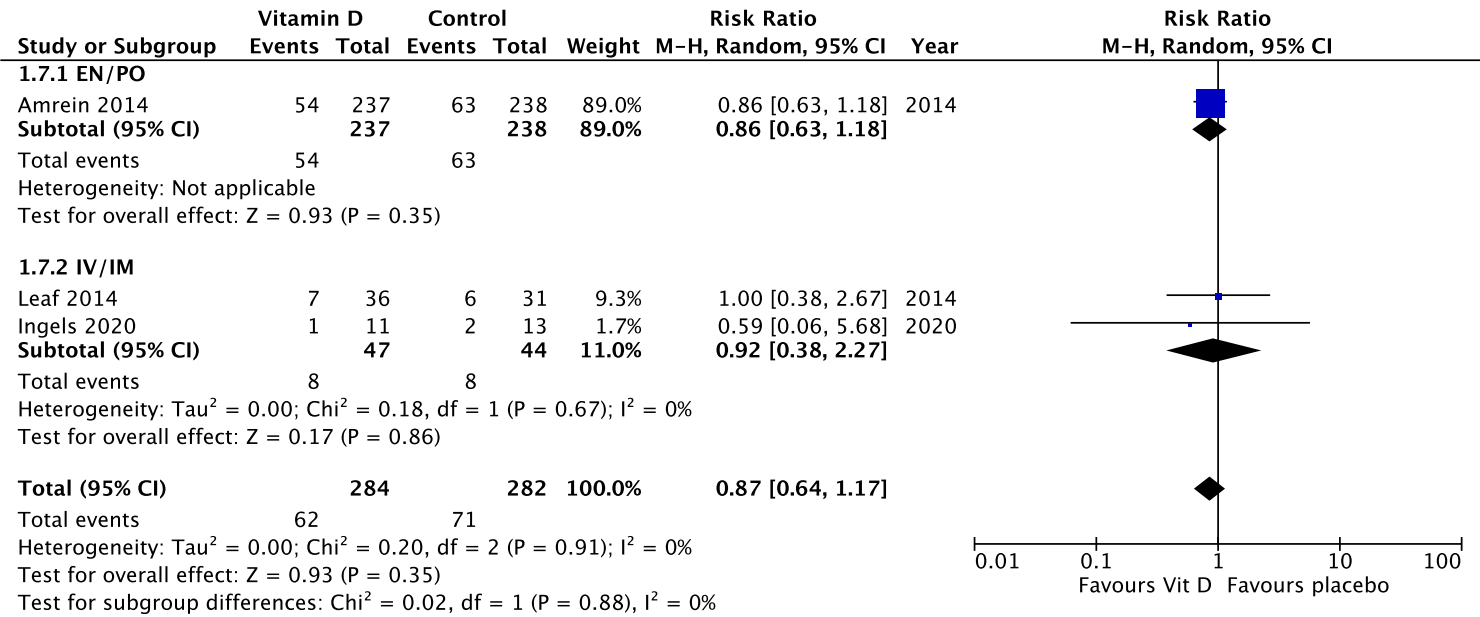

Supplement: Supplementary file 7 — Additional file 7. ICU mortality: Vitamin D compared to placebo or standard of care for critically ill patients including subgroup analysis of route of administration. [file 13054_2022_4139_MOESM7_ESM.pdf]

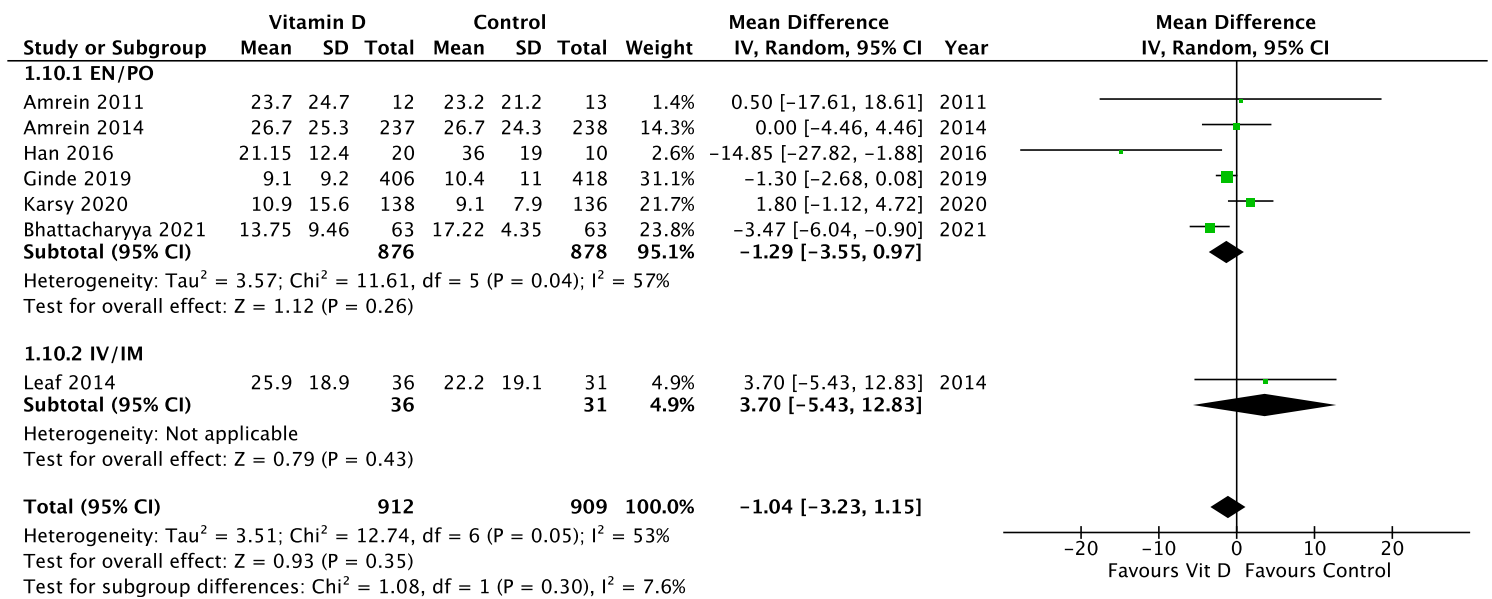

Supplement: Supplementary file 8 — Additional file 8. Hospital length of stay: Vitamin D compared to placebo or standard of care for critically ill patients including subgroup analysis of route of administration. [file 13054_2022_4139_MOESM8_ESM.pdf]

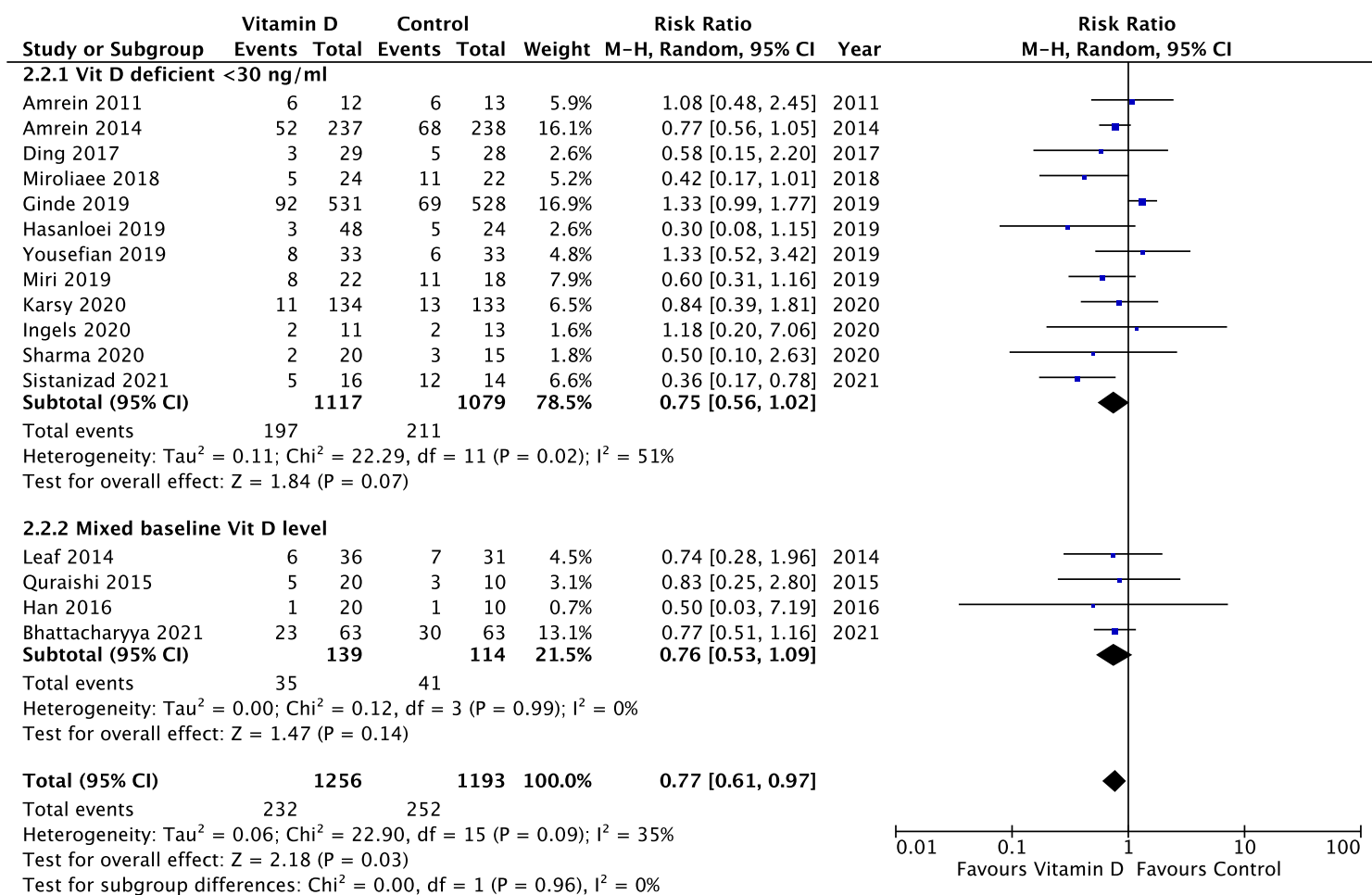

Supplement: Supplementary file 9 — Additional file 9. Overall mortality: Vitamin D compared to placebo or standard of care for critically ill patients including subgroup analysis of baseline vitamin D. [file 13054_2022_4139_MOESM9_ESM.pdf]

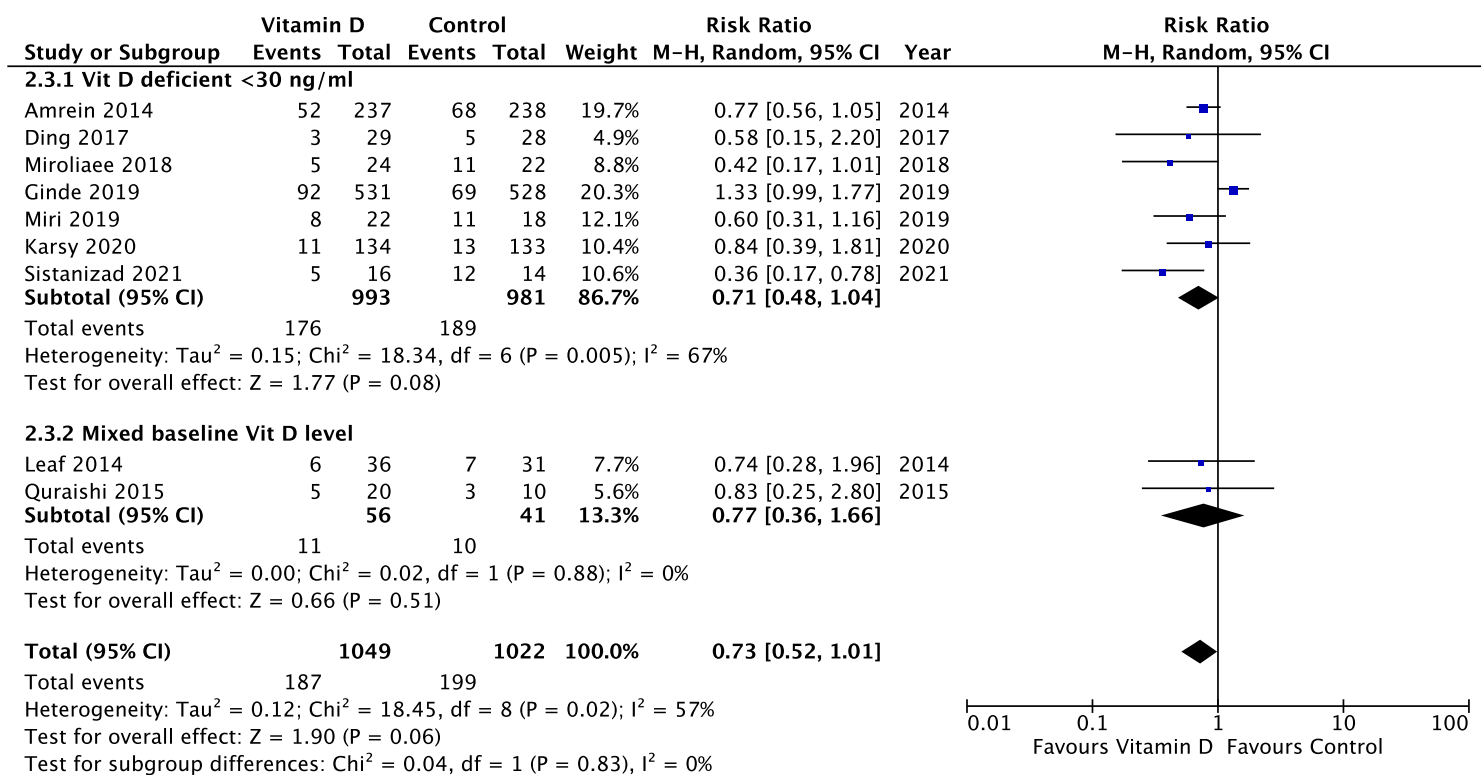

Supplement: Supplementary file 10 — Additional file 10. 28-day mortality: Vitamin D compared to placebo or standard of care for critically ill patients including subgroup analysis of baseline vitamin D. [file 13054_2022_4139_MOESM10_ESM.pdf]

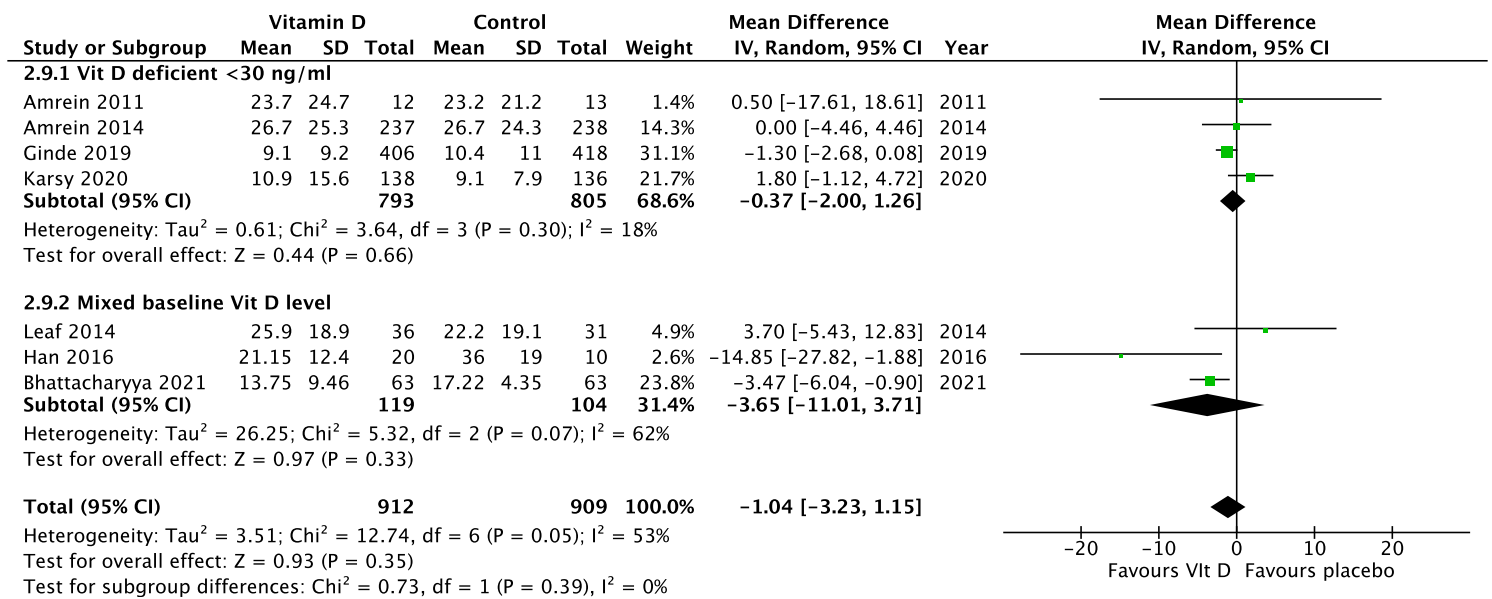

Supplement: Supplementary file 11 — Additional file 11. Hospital length of stay: Vitamin D compared to placebo or standard of care for critically ill patients including subgroup analysis of baseline vitamin D. [file 13054_2022_4139_MOESM11_ESM.pdf]

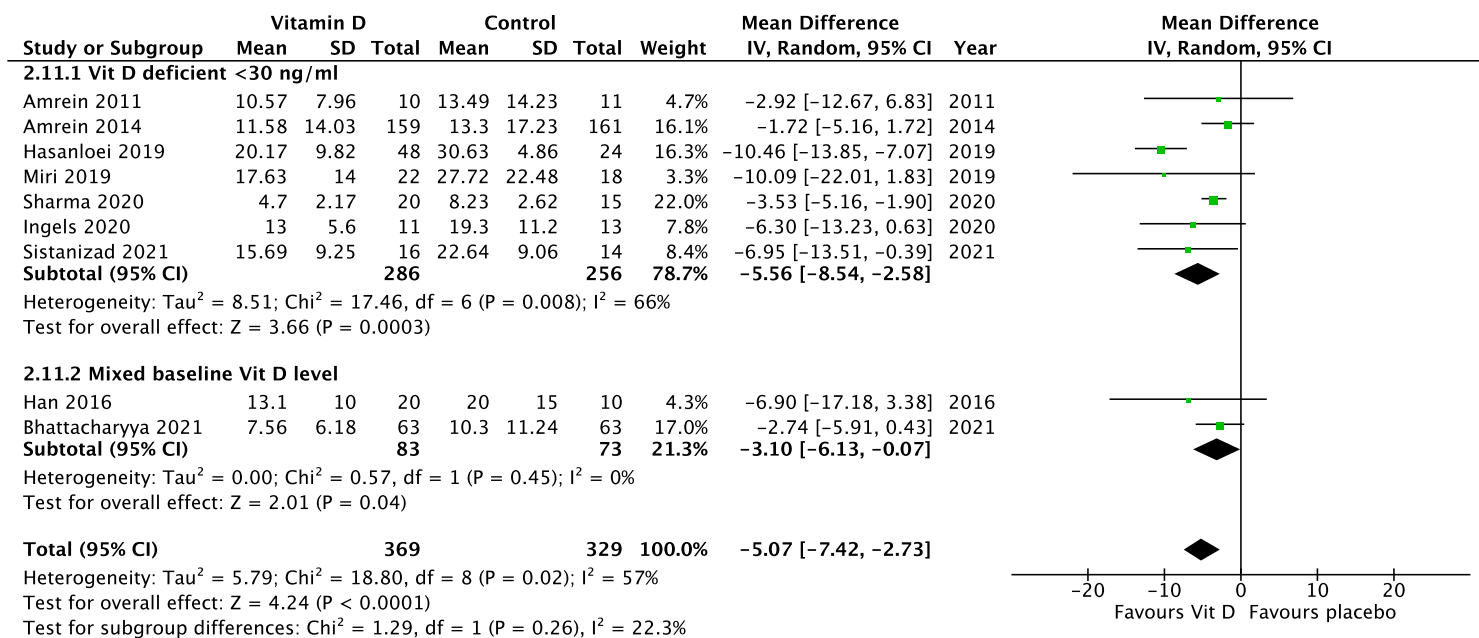

Supplement: Supplementary file 12 — Additional file 12. Duration of mechanical ventilation: Vitamin D compared to placebo or standard of care for critically ill patients including subgroup analysis of baseline vitamin D. [file 13054_2022_4139_MOESM12_ESM.pdf]
